# Supplementary material for: The respective activation and silencing of striatal direct and indirect pathway neurons support behavior encoding
Source: Nat Commun. 2023 Aug 17;14:4982. doi: 10.1038/s41467-023-40677-0 (PMC10435545; doi:10.1038/s41467-023-40677-0)
Supplement: Supplementary file 1 — Supplementary Information [file 41467_2023_40677_MOESM1_ESM.pdf]

Supplementary Information for

The respective activation and silencing of striatal direct and indirect pathway neurons support behavior encoding

Christophe Varin, Amandine Cornil, Delphine Houtteman, Patricia Bonnavion, Alban de Kerchove d'Exaerde*

Université Libre de Bruxelles (ULB), ULB Neuroscience Institute, Neurophysiology Laboratory; Brussels, Belgium
*Corresponding author (adekerch@ulb.ac.be)

This PDF includes:

Supplementary Figures 1 to 19
Supplementary Table 1
Supplementary References

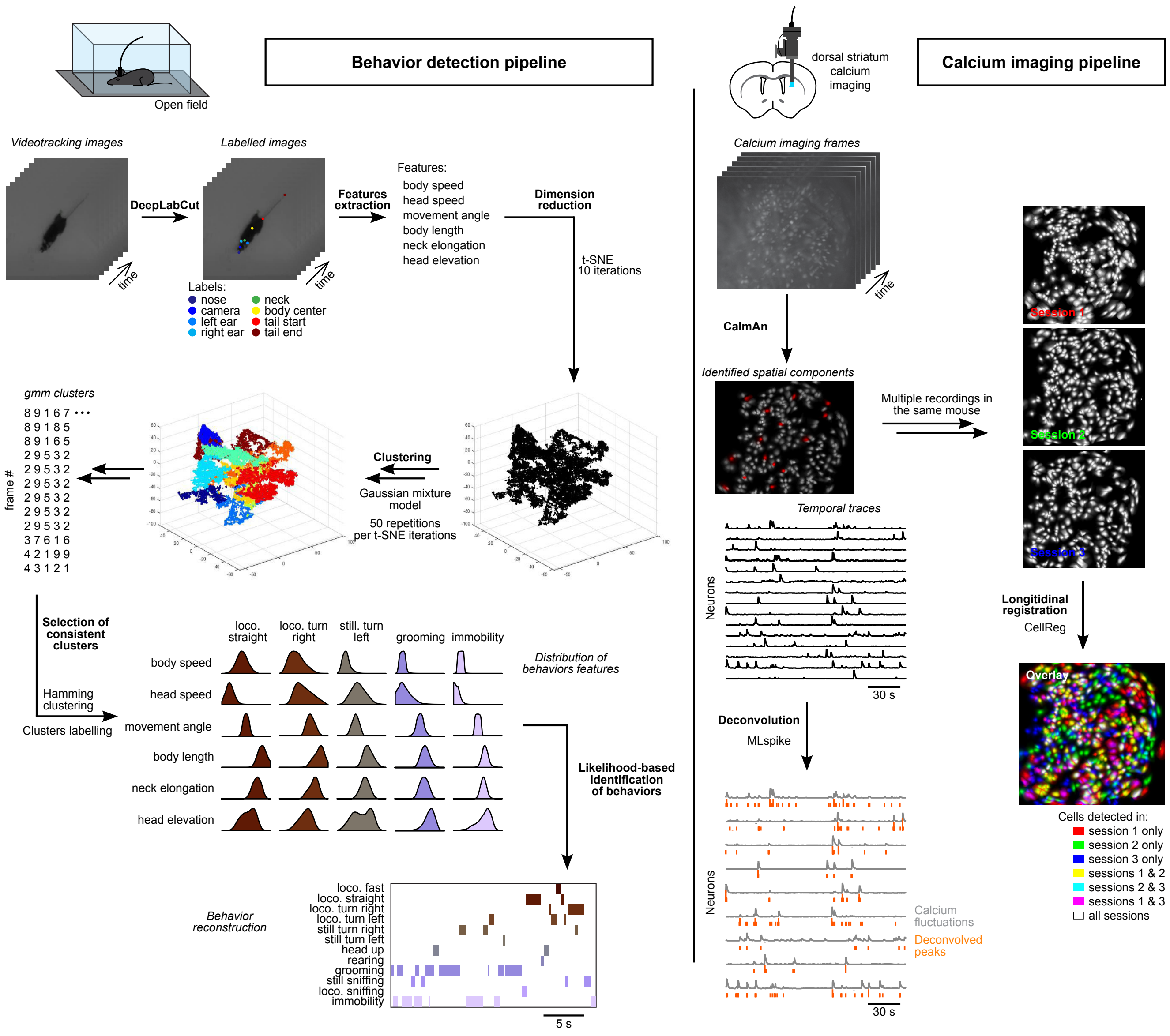

Supplementary Figure 1: General pipeline for behavioral and calcium data extraction. The synchronous video tracking and microendoscope recordings were analyzed as follows. For video tracking recordings, images are first processed using DeepLabCut to identify and track the position of 8 body parts (nose, camera, ear left, ear right, neck, body center, tail start, and tail end). Using these points, 6 features describing the animal's posture are computed and fed into multiple iterations of t-SNE dimension reduction and clustering using a Gaussian mixture model. The resulting set of clusters is clustered (Hamming distance) to identify and isolate consistent behavioral clusters and define their corresponding distributions in the feature space. These distributions are used to label all video tracking frames using likelihood-based estimators. In parallel, calcium imaging videos are first processed using CaImAn to separate and identify neurons and extract the temporal evolution of the calcium signal. This signal is then deconvolved using the MLspike algorithm. The cells that are identified during different recording sessions in the same mouse are aligned and longitudinally registered using CellReg.

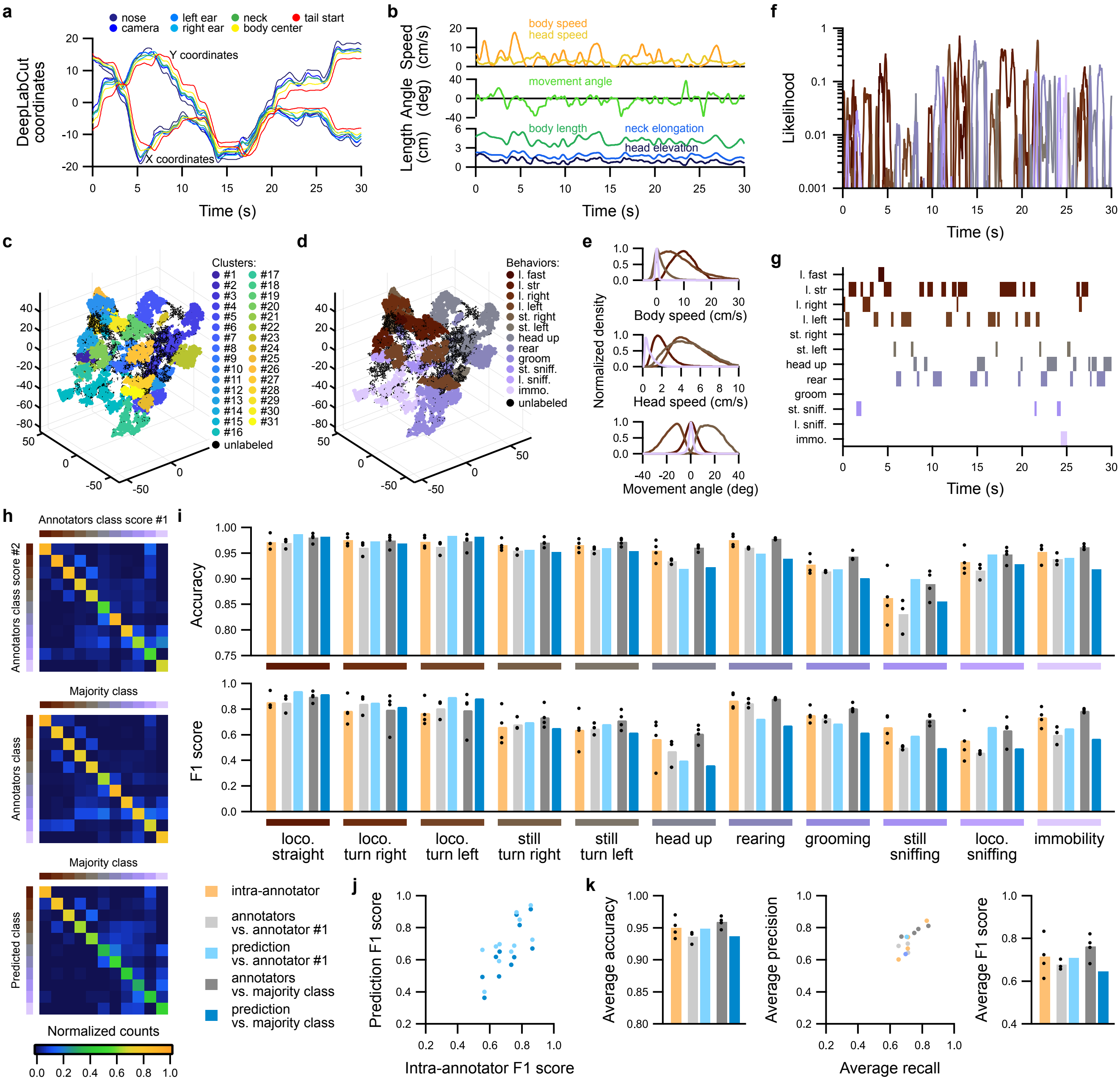

Supplementary Figure 2: Identification of mice behavior in the open field.

a-g, Example from a representative recording session of the behavior segmentation pipeline. After extracting the x-y coordinates of different mouse body parts using DeepLabCut (**a**), and their conversion into six behavioral features (**b**), the algorithm performs multiple iterations of non-linear transformations (t-SNE) to retain postural time series in a low-dimension action space followed by clustering (Gaussian mixture model) to identify groups of postures consistently clustered together forming postural archetypes (**c**). These postural archetypes are manually registered into one of the 12 behaviors capturing the behavioral repertoire mice exhibit in the open field (**d**). The distributions of the behaviors clusters in the feature space (illustrated for locomotion straight, locomotion turn left, still turn right, and immobility (**e**) are used to estimate for each time point its likelihood of belonging to each behavior cluster (**f**). The behavior was determined according to the highest likelihood (**g**). Abbreviations: l., locomotion; st., still; immo., immobility.

h, Confusion matrices comparing the annotations from the same annotator between two presentations of the same video clips (top panel), comparing classes selected by individual

annotators to the classes selected by at least half of them (majority class) (middle panel), comparing the majority class to the behaviors predicted by the behavior segmentation pipeline (bottom panel).

i, Accuracy (top panel) and F1 score (bottom panel) for each behavior computed to estimate the intra-annotator consistency between two successive annotations of the same behaviors (yellow), and performances of human annotators or predictions from the behavior segmentation pipeline relative to annotator #1 (light grey or light blue, respectively) and relative to the group majority class (dark grey or dark blue, respectively). Abbreviation: loco., locomotion.

j, The quality of the prediction behavior segmentation pipeline is correlated with the intra-annotator consistency as illustrated by the comparison for each behavior (dots) of the intra-annotator F1 score and F1 scores for the predictions relative to annotator #1 (light blue) and relative to the group majority class (dark blue).

k, Average accuracy (left panel), average precision–recall plots (middle panel), and average F1 score (right panel) combining all behaviors.

Data are presented as mean values ± SEM. Source data are provided as a Source Data file.

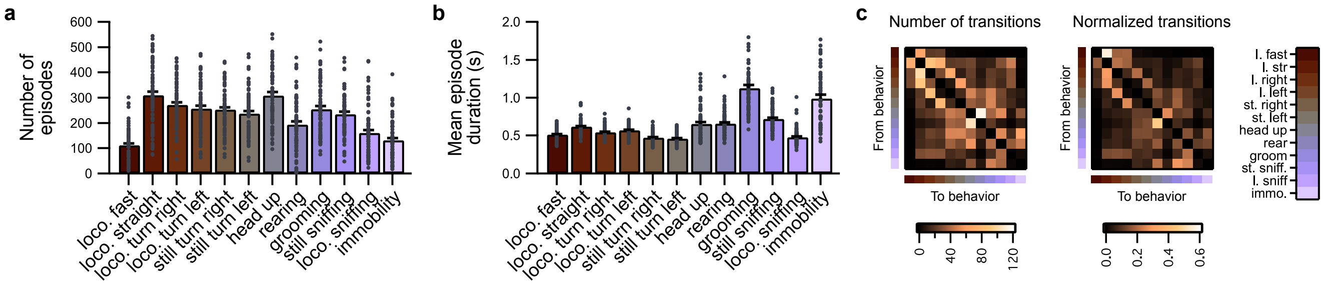

Supplementary Figure 3: Quantification of mice behavior in the open field.

a-c, Description of mouse behavior architecture during 30 min of undisturbed exploration in an open field (n = 73 sessions in 17 mice), evaluated according to the number of episodes of each behavior (**a**), the average duration of behavior episodes (**b**), and the sequential organization of behaviors, as illustrated by the averaged matrix of the number of transitions (**c**, left panel) from one behavior (lines) to a different behavior (columns), and the same normalized matrix (**c**) right panel), to evaluate the probability when stopping one behavior to begin any of the other 11 behaviors. Abbreviations: l. or loco., locomotion; st., still; immo., immobility.

Data are presented as mean values ± SEM. Source data are provided as a Source Data file.

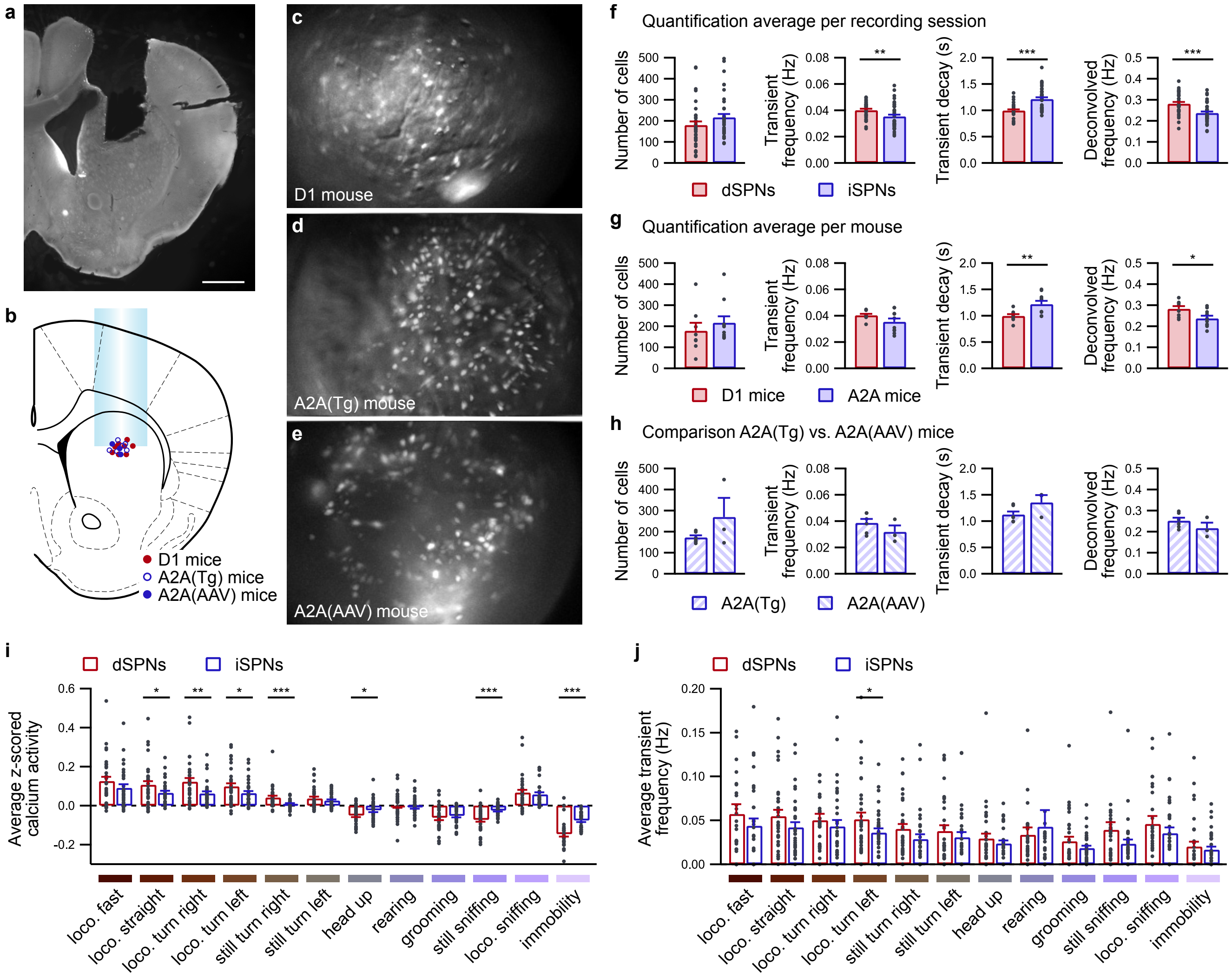

Supplementary Figure 4: One-photon calcium imaging analyses during open field exploration.

a, Photomicrograph of a coronal section from the brain of a mouse implanted with a GRIN lens in the striatum (scale bar: 1 mm). Image is representative of 17 mice.

b, Reconstruction of the GRIN lens positions for all D1 mice (n = 8) and A2A mice (A2A(Tg), n = 6; A2A(AAV), n = 3).

c-e, Representative image of the maximum fluorescence intensity projection of SPNs labeled with GCaMP6s in a D1 mouse (**c**), A2A(Tg) mouse (**d**), and A2A(AAV) mouse (**e**).

f, Quantification for each recording session of the identified neuron number, calcium transient average frequency, average transient decay characteristic time, and deconvolved calcium activity for dSPNs (red; n = 33 sessions in 8 mice) and iSPNs (blue; n = 40 sessions in 9 mice) (permutation-based two-sided t-test, dSPNs vs. iSPNs: number of cells, p = 0.101; transient frequency, ** p = 0.0062; transient decay, *** p = 0; deconvolved activity, *** p = 0).

g, Same as **f**, averaged per mouse (D1 mice, n = 8; A2A mice, n = 9) (permutation-based two-sided t-test, D1 vs. A2A: number of cells, p = 0.220; transient frequency, p = 0.069; transient decay, ** p = 0.0078; deconvolved activity, * p = 0.0118).

h, Comparison of the above parameters, targeting GCaMP6s expression in iSPNs using transgenic reporter mice (A2A(Tg), n = 6) or AAV injection (A2A(AAV), n = 3). No significant difference was observed between A2A(Tg) mice and A2A(AAV) mice (permutation-based two-sided t-test,

A2A(Tg) vs. A2A(AAV): number of cells, $p = 0.1076$; transient frequency, $p = 0.1094$; transient decay, $p = 0.0738$; deconvolved activity, $p = 0.11$).
i-j, Average population activity during each behavior evaluated from z-scored calcium signal (**i**) and calcium transients peaks (**j**) (threshold crossing) for dSPNs (red; n = 33 sessions in 8 mice) and iSPNs (blue; n = 40 sessions in 9 mice) (linear mixed effect model followed by post-hoc permutation-based two-sided t-test, dSPNs vs. iSPNs: * $p < 0.05$, ** $p < 0.01$, *** $p < 0.001$). Abbreviation: loco., locomotion.
Data are presented as mean values ± SEM. Detailed statistics are displayed in Supplementary Table 1. Source data are provided as a Source Data file.

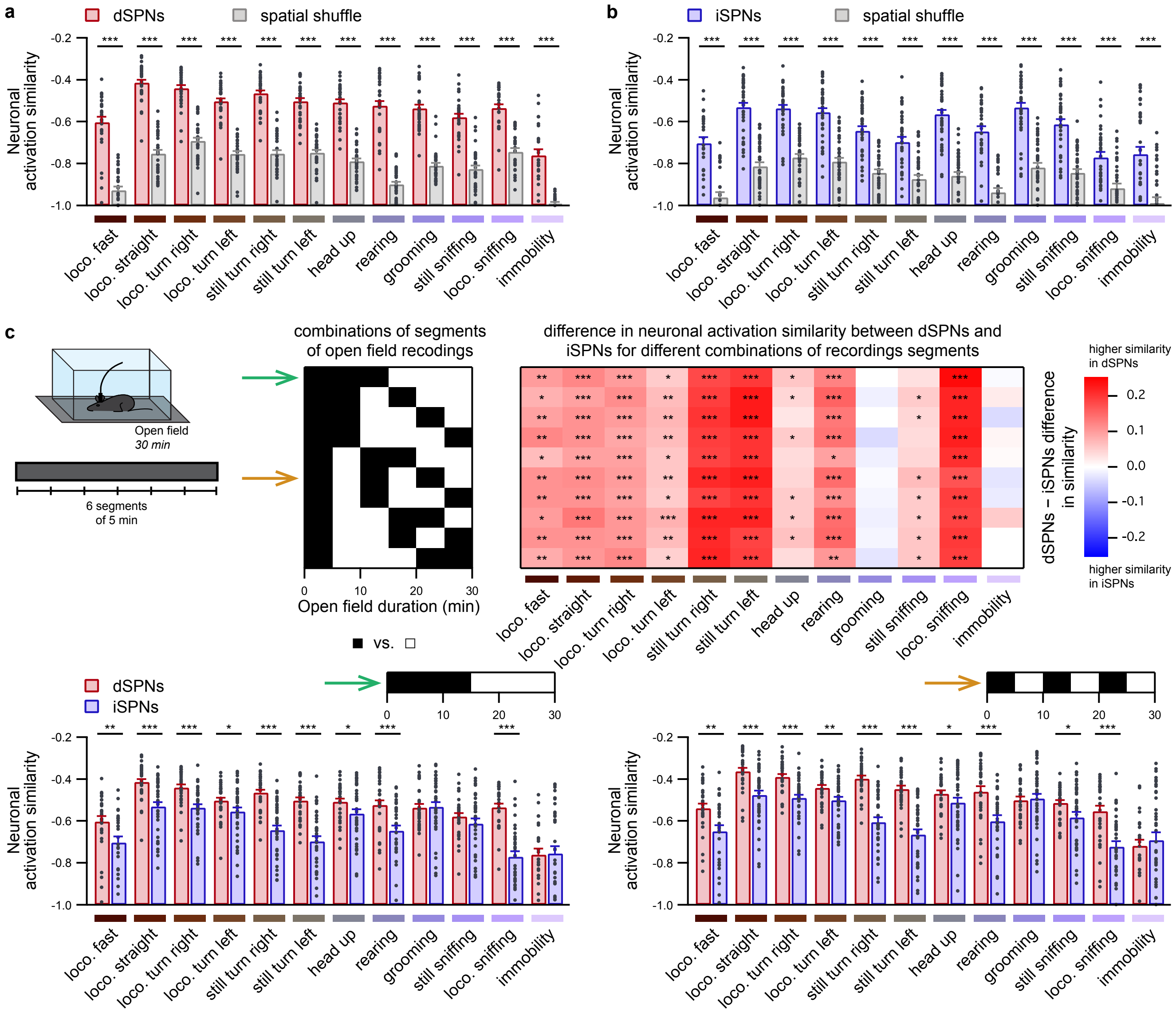

Supplementary Figure 5: Time invariance of differences in neuronal activation similarity between dSPNs and iSPNs.

a-b, Neuronal activation similarity between the first and second halves of open field exploration in dSPNs (**a**) (red; n = 33 sessions in 8 mice) and iSPNs (**b**) (blue; n = 40 sessions in 9 mice) compared with the spatial shuffle of neurons between the first and second halves of open field exploration (gray bars) (linear mixed effect model followed by post-hoc permutation-based two-sided t-test, dSPNs or iSPNs vs. shuffle: *** $p < 0.001$). Abbreviation: loco., locomotion.

c, The 30 min open field recording is split into 6 segments lasting 5 min each (top left panel), and the difference between the dSPN and iSPN neuronal activation similarities (dSPNs: red, n = 33 sessions in 8 mice; iSPNs: blue, n = 40 sessions in 9 mice) is calculated for all combinations of 5 min segments into two 15 min segments (3 segments per groups) (top middle and right), highlighting that the difference in similarity between dSPNs and iSPNs is a time-invariant property of the neuronal code. (Bottom) Detailed representation of neuronal activation similarity for two splitting schemes, indicated by colored arrows (linear mixed effect model followed by post-hoc permutation-based two-sided t-test, dSPNs vs. iSPNs: * $p < 0.05$, ** $p < 0.01$, *** $p < 0.001$). Abbreviation: loco., locomotion.

Data are presented as mean values ± SEM. Detailed statistics are displayed in Supplementary Table 1. Source data are provided as a Source Data file.

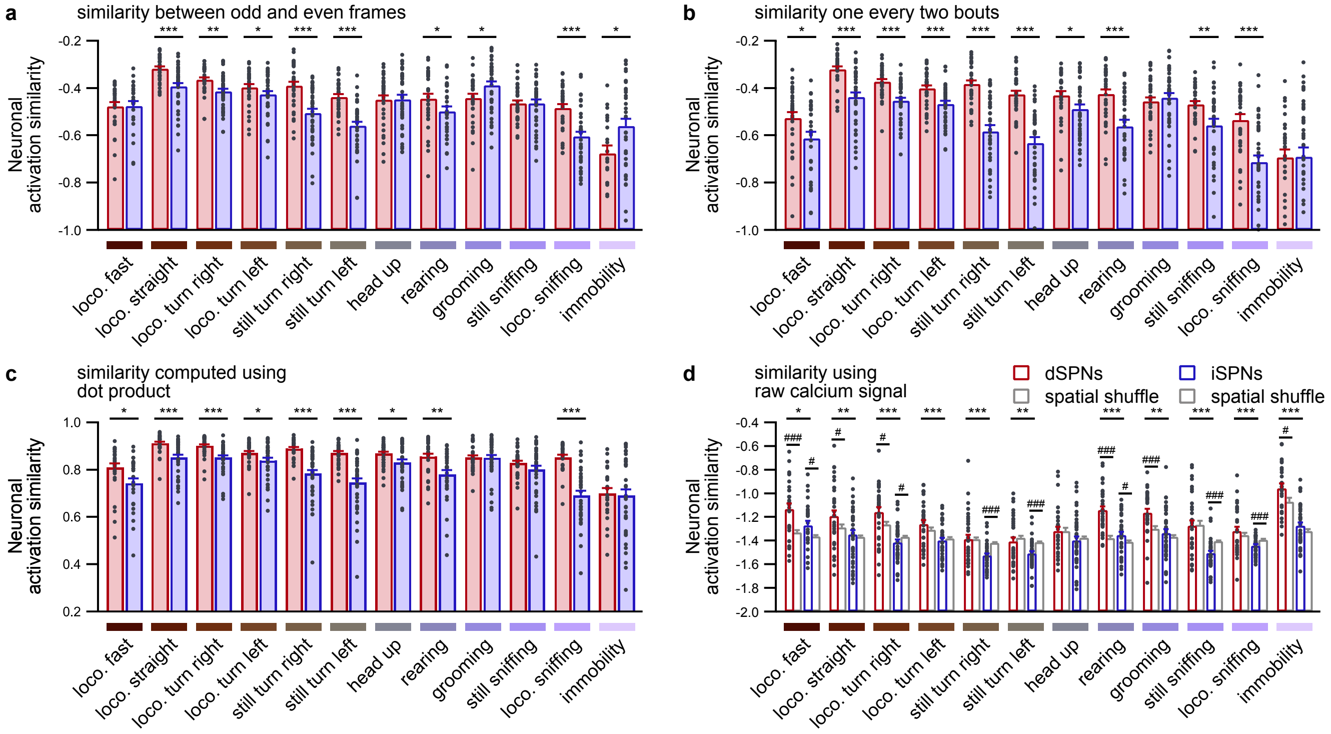

Supplementary Figure 6: Additional controls related to the computation of neuronal activation similarity between dSPNs and iSPNs.

a, Neuronal activation similarity between dSPNs (red; n = 33 sessions in 8 mice) and iSPNs (blue; n = 40 sessions in 9 mice) for each behavior, as computed by comparing odd and even frames (linear mixed effect model followed by post-hoc permutation-based two-sided t-test, dSPNs vs. iSPNs: * $p < 0.05$, ** $p < 0.01$, *** $p < 0.001$).

b, Neuronal activation similarity between dSPNs (red; n = 33 sessions in 8 mice) and iSPNs (blue; n = 40 sessions in 9 mice), as computed by comparing one episode out of two for each behavior to complementary episodes (linear mixed effect model followed by post-hoc permutation-based two-sided t-test, dSPNs vs. iSPNs: * $p < 0.05$, ** $p < 0.01$, *** $p < 0.001$).

c, Neuronal activation similarity between the first and second halves of open field exploration between dSPNs (red; n = 33 sessions in 8 mice) and iSPNs (blue; n = 40 sessions in 9 mice) calculated using the dot product of the two neuronal activity vectors (linear mixed effect model followed by post-hoc permutation-based two-sided t-test, dSPNs vs. iSPNs: * $p < 0.05$, ** $p < 0.01$, *** $p < 0.001$).

d, Neuronal activation similarity between the first and second halves of open field exploration between dSPNs (red; n = 33 sessions in 8 mice) and iSPNs (blue; n = 40 sessions in 9 mice) and compared with the spatial shuffle of neurons between the first and second halves of open field exploration (gray bars) evaluated using raw fluorescence signal (linear mixed effect model followed by post-hoc permutation-based two-sided t-test, dSPNs vs. iSPNs: * $p < 0.05$, ** $p < 0.01$, *** $p < 0.001$; dSPNs or iSPNs vs. shuffle: $^{\#}$ $p < 0.05$, $^{\#\#}$ $p < 0.01$, $^{\#\#\#}$ $p < 0.001$).

Abbreviation: loco., locomotion.

Data are presented as mean values ± SEM. Detailed statistics are displayed in Supplementary Table 1. Source data are provided as a Source Data file.

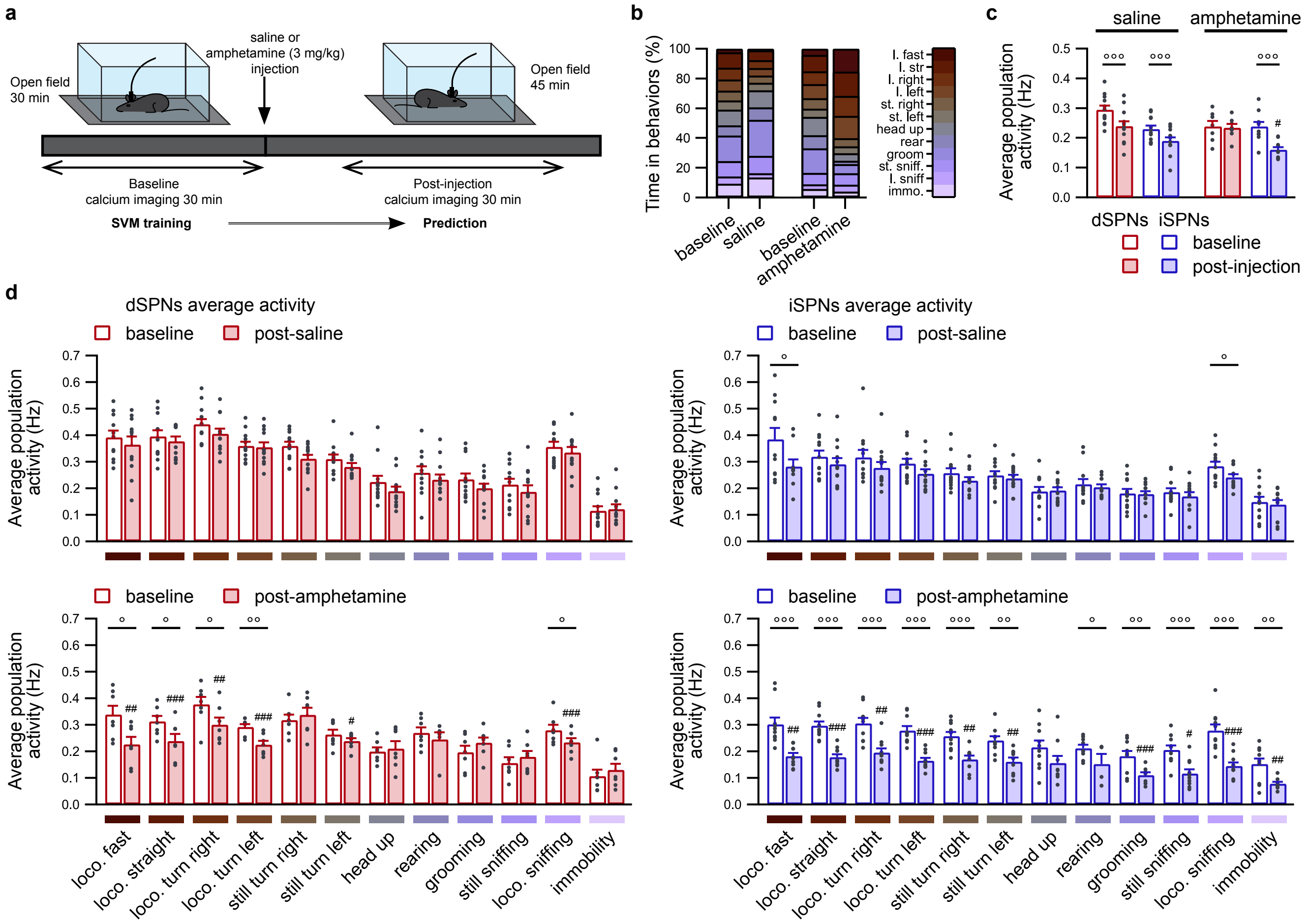

Supplementary Figure 7: Amphetamine administration modulates behavior distribution and population activity in SPNs.

a, Mice expressing GCaMP6s in either dSPNs or iSPNs freely explored a well-known open field for 30 min (baseline period), received an injection of either saline or amphetamine (3 mg/kg), and were placed back into the open field for an additional 45 min. The post-injection period began 10 min after the injection and lasted 30 min. The SVM classifiers were trained on the baseline period, and the predictions were performed based on the neuronal activity in the post-injection period.

b, Average distribution of behaviors over 30 min of baseline and 30 min post-saline or post-amphetamine administration in mice expressing GCaMP6s in either dSPNs or iSPNs (saline: n = 23 sessions in 17 mice; amphetamine: n = 15 sessions in 15 mice). Abbreviations: l., locomotion; st., still; immo., immobility.

c, Average population activity for dSPNs (red; saline: n = 12 sessions in 8 mice; amphetamine: n = 7 sessions in 7 mice) and iSPNs (blue; saline: n = 11 sessions in 9 mice; amphetamine: n = 8 sessions in 8 mice) during 30 min of baseline (unfilled bars) and 30 min following saline or amphetamine administration (colored bars) (linear mixed effect model followed by post-hoc permutation-based two-sided t-test, baseline vs. post injection: °°° $p < 0.001$; saline vs. amphetamine: $^{\#}$ $p < 0.05$).

d, Average population activity recorded for dSPNs (left panels) and iSPNs (right panels) during baseline and after saline injection (top panels; dSPNs: n = 12 sessions in 8 mice; iSPNs: n = 11 sessions in 9 mice) or amphetamine injection (bottom panels; dSPNs: n = 7 sessions in 7 mice; iSPNs: n = 8 sessions in 8 mice) (linear mixed effect model followed by post-hoc

permutation-based two-sided t-test, baseline vs. post-injection: ° $p < 0.05$, °° $p < 0.01$, °°° $p < 0.001$; saline vs. amphetamine: # $p < 0.05$, ## $p < 0.01$, ### $p < 0.001$). Abbreviation: loco., locomotion.

Data are presented as mean values ± SEM. Detailed statistics are displayed in Supplementary Table 1. Source data are provided as a Source Data file.

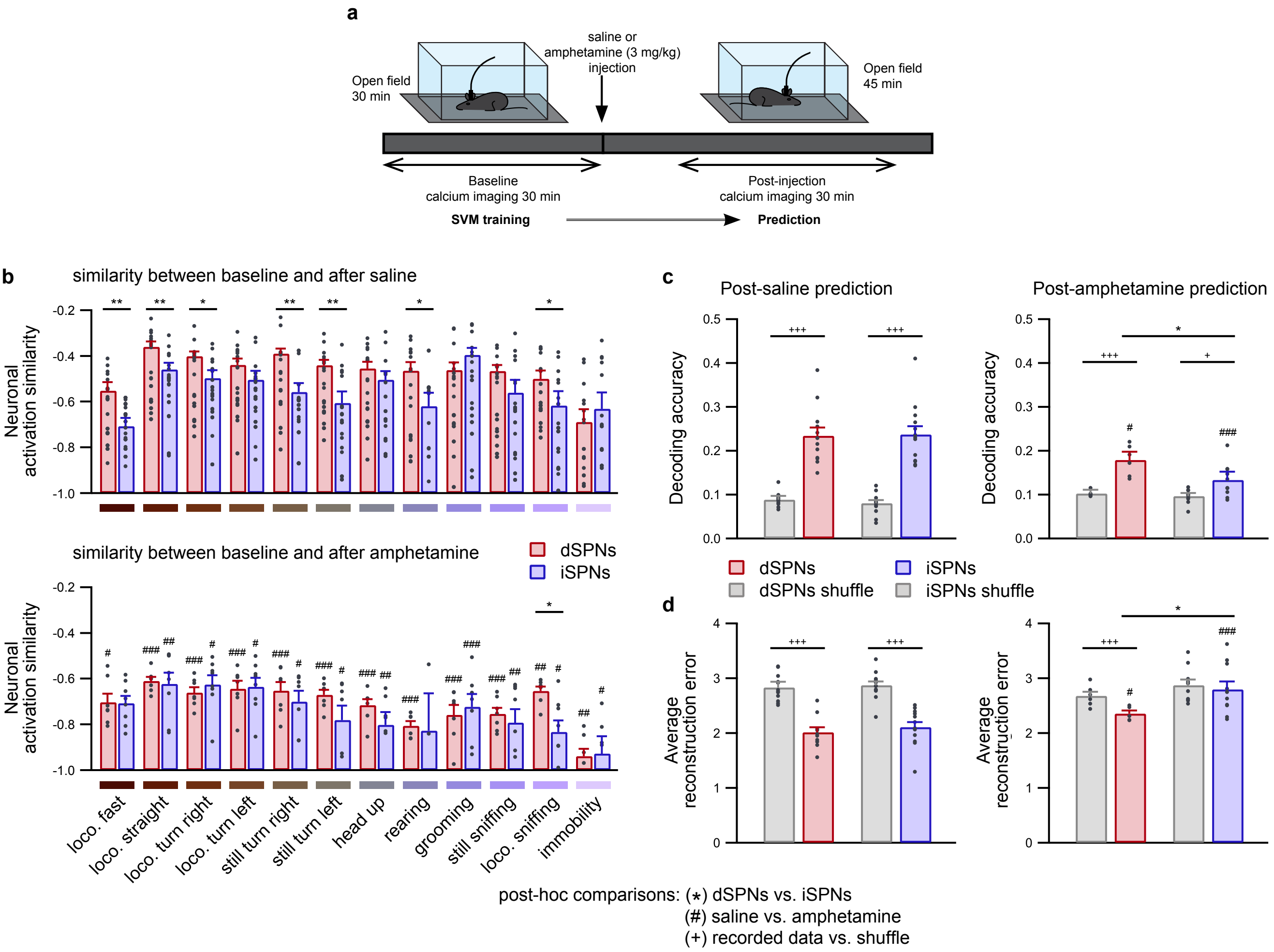

Supplementary Figure 8: Amphetamine administration reduces the neuronal activation similarity of dSPNs and iSPNs and disrupts SVM-based behavior predictions.

a, Mice expressing GCaMP6s in either dSPNs or iSPNs freely explored a well-known open field for 30 min (baseline period), received an injection of either saline or amphetamine (3 mg/kg), and were placed back into the open field for an additional 45 min. The post-injection period began 10 min after the injection and lasted 30 min. The SVM classifiers were trained on the baseline period, and the predictions were performed based on the neuronal activity in the post-injection period.

b, Neuronal activation similarity between baseline and postinjection periods of open field exploration in dSPNs (red) and iSPNs (blue) for all detected behaviors following saline (top; dSPNs: n = 12 sessions in 8 mice; iSPNs: n = 11 sessions in 9 mice) or amphetamine (3 mg/kg) injection (bottom; dSPNs: n = 7 sessions in 7 mice; iSPNs: n = 8 sessions in 8 mice) (linear mixed effect model followed by post-hoc permutation-based two-sided t-test, dSPNs vs. iSPNs: * $p < 0.05$, ** $p < 0.01$, *** $p < 0.001$; saline vs. amphetamine: $^{\#}$ $p < 0.05$, $^{\#\#}$ $p < 0.01$, $^{\#\#\#}$ $p < 0.001$). Abbreviation: loco., locomotion.

c-d, Accuracy of behavior prediction (**c**) and mean behavioral reconstruction error (**c**) using dSPNs (red) or iSPNs (blue) after saline (left panels; dSPNs: n = 10 sessions in 6 mice; iSPNs: n = 13 sessions in 9 mice) or amphetamine administration (right panels; dSPNs: n = 6 sessions in 6 mice; iSPNs: n = 9 sessions in 9 mice), as compared with time-lagged data (gray) (linear mixed effect model followed by post-hoc permutation-based two-sided t-test, dSPNs vs. iSPNs:

* $p < 0.05$; saline vs. amphetamine: $^{\#}$ $p < 0.05$, $^{\#\#}$ $p < 0.01$, $^{\#\#\#}$ $p < 0.001$; dSPNs or iSPNs vs. shuffle: $^{+}$ $p < 0.05$; $^{+++}$ $p < 0.001$).
Data are presented as mean values ± SEM. Detailed statistics are displayed in Supplementary Table 1. Source data are provided as a Source Data file.

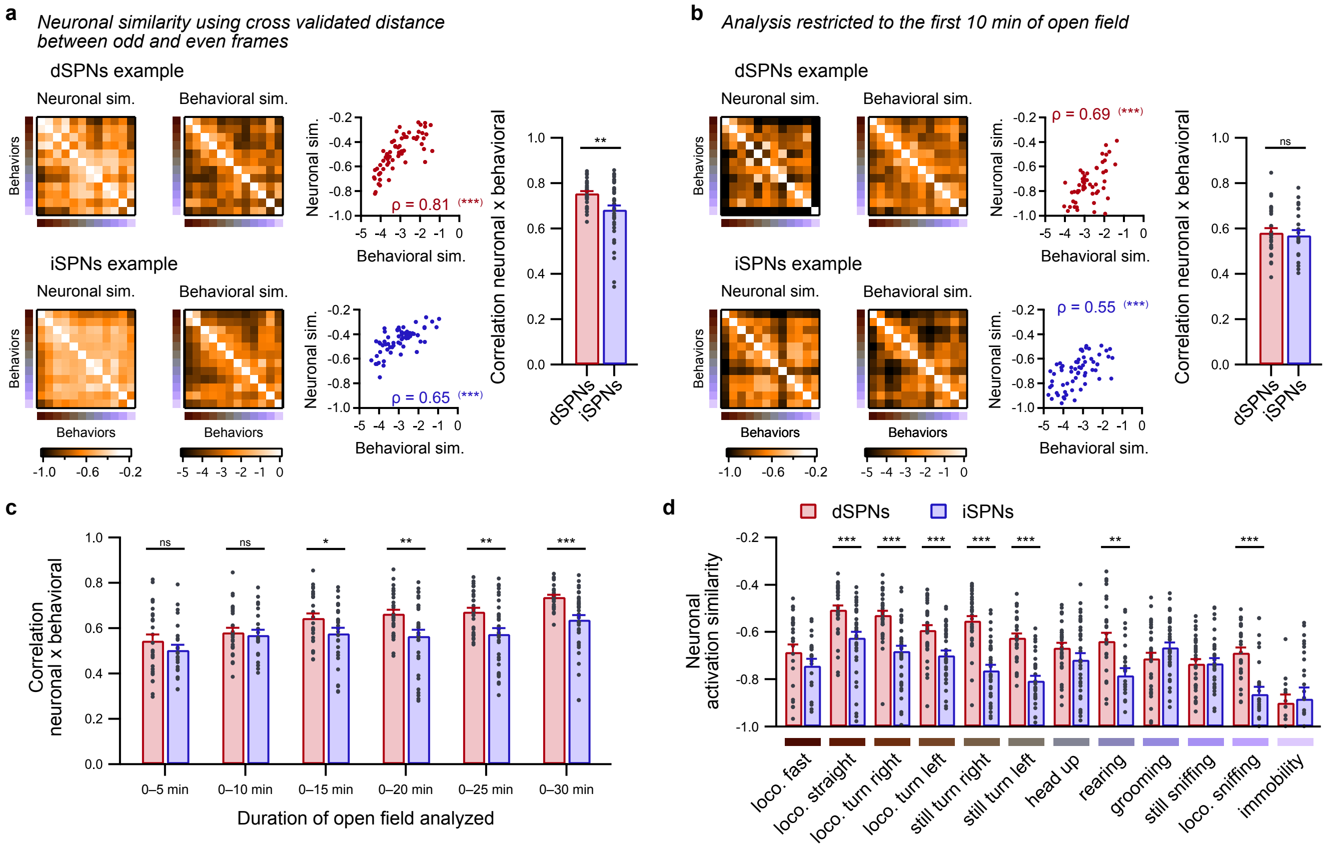

Supplementary Figure 9: Complements related to correlations between pairwise neuronal similarity and behavioral similarity between behaviors.

a, Examples of matrices of pairwise neuronal activation similarity between behaviors computed using the cross-validated Euclidean distance between odd and even frames, matrices of pairwise behavioral similarity between behaviors (left panels), and the corresponding scatterplot for pairs of behaviors (middle; Spearman correlation two-sided, dSPNs, *** p = 1.1e-31; iSPNs, *** p = 3.5e-17) for one session from one representative D1 mouse (top) and one representative D2 mouse (bottom). The average correlation coefficient (right) between the pairwise behavioral and neuronal similarities was higher for dSPNs (red; n = 33 sessions in 8 mice) than for iSPNs (blue; n = 40 sessions in 9 mice) (permutation-based two-sided t-test, dSPNs vs. iSPNs: *** p = 0.0026). The results are similar to those displayed in Fig. 3c-f.

b, Same as a, with both the neuronal and behavioral similarities computed for only the first 10 min of open field exploration. In this condition, the average correlation between the behavioral and neuronal similarities was similar for dSPNs (red; n = 24 sessions in 8 mice) and iSPNs (blue; n = 21 sessions in 9 mice) (middle; Spearman correlation two-sided, dSPNs, *** p = 3.8e-20; iSPNs, *** p = 5.8e-12) (right; permutation-based two-sided t-test, dSPNs vs. iSPNs: ns p > 0.05).

c, Comparison of correlation coefficients between behavioral and neuronal similarities evaluated for different durations of open field exploration in increments of 5 min for dSPNs (red; n = 33 sessions in 8 mice) and iSPNs (blue; n = 40 sessions in 9 mice) (linear mixed effect model followed by post-hoc permutation-based two-sided t-test, dSPNs vs. iSPNs: ns p > 0.05; * p < 0.05, ** p < 0.01, *** p < 0.001).

d, Neuronal activation similarity between the first 5 min and subsequent 5 min of open field exploration remained higher in dSPNs (red; n = 24 sessions in 8 mice) than in iSPNs (blue; n = 21 sessions in 9 mice) during locomotion (loco.) straight, locomotion turn right and left,

still turn right and left, rearing, and locomotion sniffing (linear mixed effect model followed by post-hoc permutation-based two-sided t-test, dSPNs vs. iSPNs: * $p < 0.05$, ** $p < 0.01$, *** $p < 0.001$).
Data are presented as mean values ± SEM. Detailed statistics are displayed in Supplementary Table 1. Source data are provided as a Source Data file.

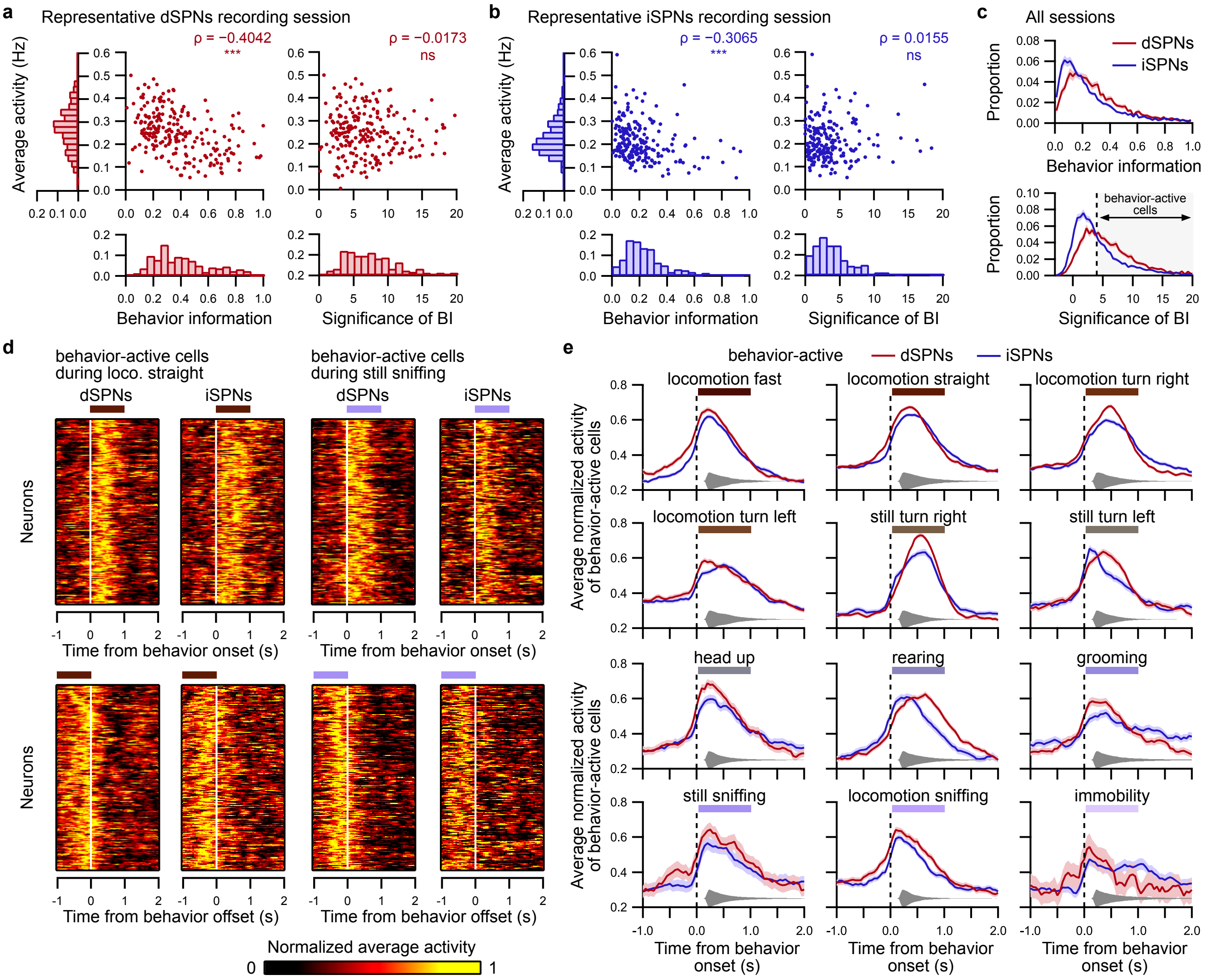

Supplementary Figure 10: Significance of mutual information and activity of behavior-active cells.

a-b, Scatterplots of the relationship between the behavior information (left) or significance of the behavior information (right) and the average activity of dSPNs (**a**) and iSPNs (**b**) in one representative session. Note the significant inverse correlation (Spearman correlation: ns $p > 0.05$, *** $p < 0.001$) between the behavior information and event rate, which indicates that the behavior information is biased toward neurons firing at a low rate. Using the significance of the behavior information, which is computed by comparing the observed value of the behavior information to random shuffles, removes this bias.

c, Average distribution of behavior information (top panel) and significance of behavior information (bottom panel) for dSPNs (red, n = 29 sessions in 8 mice) and iSPNs (blue, n = 37 sessions in 9 mice). Cells are identified as behavior-active when the level of significance of behavior information exceeds 4 sigma of the shuffled distribution (gray area; left panel; threshold indicated by the black dashed vertical line).

d, Representative examples of average activity of dSPNs and iSPNs classified as behavior-active during locomotion straight (left panels) or still sniffing (right panels) aligned to behavior episode onsets (top panels) or episode offsets (bottom panel). Abbreviation: loco., locomotion.

e, Average responses aligned to behavior episodes onsets for dSPNs (red) and iSPNs (blue) classified as behavior-active for each of the behaviors, and corresponding distribution of behavior episode durations (horizontal violin plot; gray).
Data are presented as mean values ± SEM. Detailed statistics are displayed in Supplementary Table 1. Source data are provided as a Source Data file.

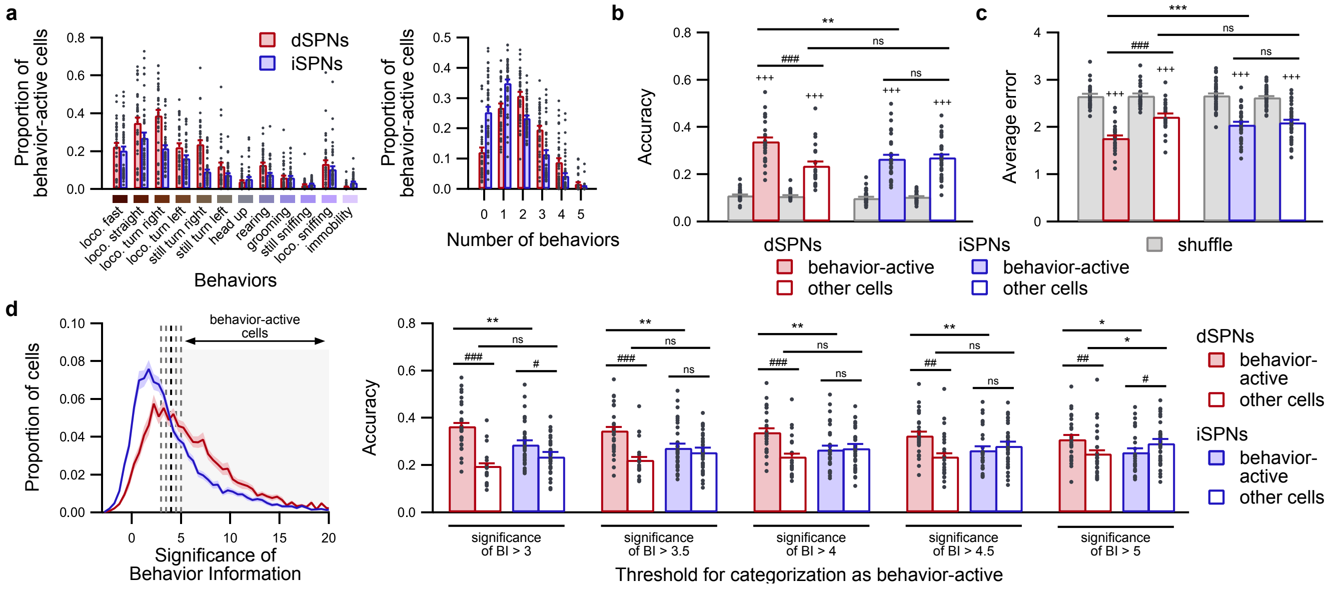

Supplementary Figure 11: Characterization of behavior-active cells and decoding performance using behavior-active cells.

a, Average fraction of dSPNs and iSPNs identified as behavior-active (significance of behavior information above 4) for each behavior (left panel) and the average fraction of dSPNs and iSPNs labeled as behavior-active during no behavior or one to five behaviors (right panel) (dSPNs: n = 29 sessions from 8 mice; iSPNs: n = 37 sessions from 9 mice). Abbreviation: loco., locomotion.

b-c, Decoding accuracy (**b**) and average reconstruction error (**c**) of SVM classifiers applied to behavior-active (plain bars) or non-behavior-active (unfilled bars) dSPNs (red; n = 29 sessions in 8 mice) or iSPNs (blue; n = 37 sessions in 9 mice), compared with the chance level when decoding classifiers trained on time-lagged data (gray bars) (linear mixed effect model followed by post-hoc permutation-based two-sided t-test, dSPNs vs. iSPNs: ns $p > 0.05$, *** $p < 0.01$; behavior-active vs. other cells: $^{\#}$ $p < 0.05$, $^{\#\#\#}$ $p < 0.001$ observed data vs. time-lagged: $^{+++}$ $p < 0.001$). Note that the decoding performance based on either behavior-active neurons or other cells is better than that based on time-shuffled data, and the decoding performance is better with behavior-active cells than with non-behavior-active cells in dSPN recordings (red bars; n = 29 sessions from 8 mice), whereas performance is similar between both cell groups in iSPNs recordings (blue bars; n = 37 sessions from 9 mice). Moreover, the decoding performance is better for behavior-active dSPNs than for behavior-active iSPNs.

d, Effect of modifying the threshold used for the level of significance of behavior information for labeling neurons as behavior-active (left panel) on the decoding accuracy (right panel) when predicting behaviors using behavior-active neurons (plain bars) or non-behavior-active neurons (unfilled bars) in dSPN (red bars; n = 29 sessions from 8 mice) and iSPN (blue bars; n = 37 sessions from 9 mice) recordings (linear mixed effect model followed by post-hoc permutation-based two-sided t-test, dSPNs vs. iSPNs: ns $p > 0.05$, ** $p < 0.01$; behavior-active vs. other cells: $^{\#}$ $p < 0.05$, $^{\#\#\#}$ $p < 0.001$). The use of 5 different thresholds (ranging from 3 to 5 SD, corresponding to the position of the actual behavior information in comparison to the distribution of the behavior information calculated from random permutations) maintains significantly higher decoding performance when using behavior-active dSPNs than when using behavior-active iSPNs.

Data are presented as mean values ± SEM. Detailed statistics are displayed in Supplementary Table 1. Source data are provided as a Source Data file.

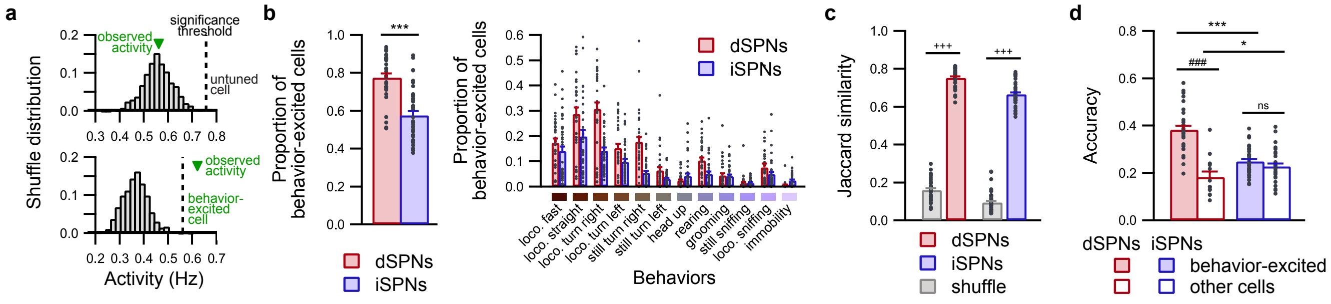

Supplementary Figure 12: Behaviors prediction using behavior-excited cells.

a, Alternatively, cells were classified as behavior-excited during one behavior when its average activity during this behavior exceeded the 0.1% percentile of the shuffle distribution of average activities obtained from random permutations (1000 replicates).

b, Proportion of behavior-excited cells (for any behavior) identified among dSPNs (n = 29 sessions in 8 mice) and iSPNs (n = 37 sessions in 9 mice) (left panel; permutation-based two-sided t-test, dSPNs vs. iSPNs: *** p = 0), and average fraction of dSPNs and iSPNs identified as behavior-excited for each behavior (right panel). Abbreviation: loco., locomotion.

c, Quantification of the similarity between the classification of cells as behavior-active (using the significance of behavior information) and the classification of cells as behavior-excited (using the shuffle procedure for each behavior) using the Jaccard similarity coefficient (linear mixed effect model followed by post-hoc permutation-based two-sided t-test, dSPNs vs. shuffle: $^{+++}$ p = 0; iSPNs vs. shuffle: $^{+++}$ p = 0).

d, Decoding accuracy of SVM classifiers applied to behavior-excited (plain bars) or non-behavior-excited (unfilled bars) dSPNs (red; n = 29 sessions in 8 mice) or iSPNs (blue; n = 37 sessions in 9 mice) (linear mixed effect model followed by post-hoc permutation-based two-sided t-test, dSPNs vs. iSPNs: * p = 0.035, *** p = 0.0002; behavior-active vs. other cells: ns p = 0.097, $^{\#\#\#}$ p = 0).

Data are presented as mean values ± SEM. Detailed statistics are displayed in Supplementary Table 1. Source data are provided as a Source Data file.

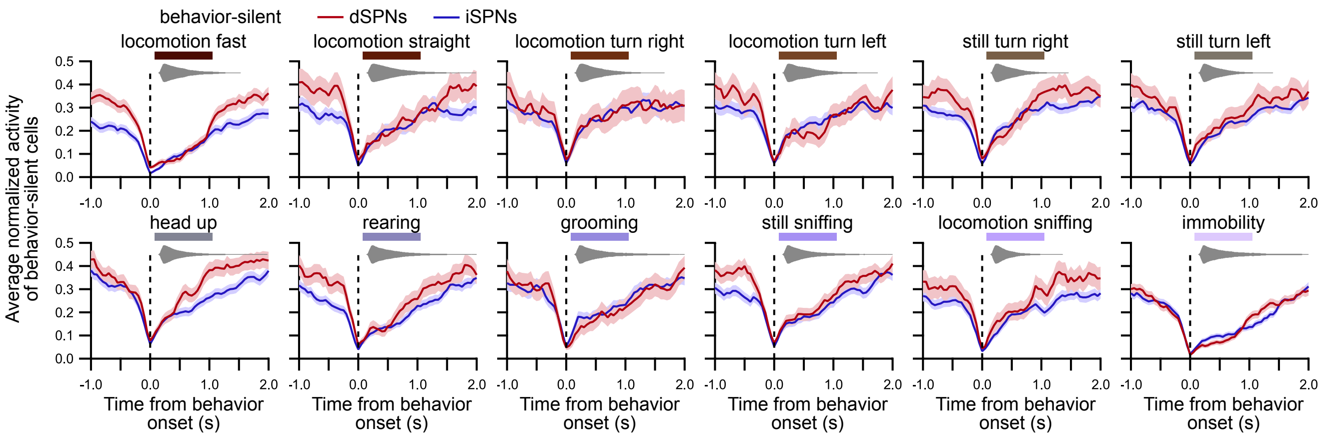

Supplementary Fig. 13: Response of behavior-silent SPNs.

Average responses aligned to behavior episodes onsets for dSPNs (red) and iSPNs (blue) classified as behavior-silent for each of the behaviors, and corresponding distribution of behavior episode durations (horizontal violin plot; gray).

Data are presented as mean values ± SEM.

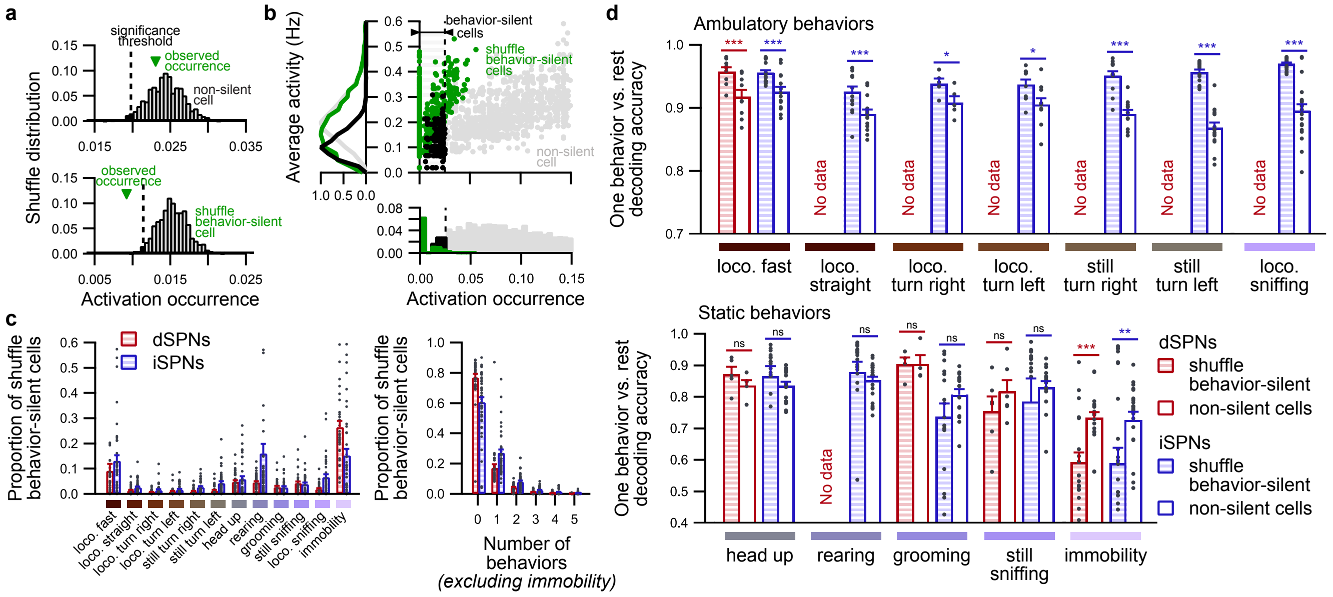

Supplementary Fig. 14: Contribution of behavior-silent cells to neural code when identified using a shuffle procedure.

a, For each cell, its activation occurrence is compared to the distribution of activation occurrences obtained from random permutations (1000 replicates). A cell is classified as shuffle behavior-silent when its observed activation occurrence is below the 5% percentile of the shuffle distribution (bottom panel); otherwise, the cell is classified as non-silent (top panel).

b, Scatterplot of the relationship between the activation occurrence and the average activity of SPNs and corresponding histogram of activation occurrence values (bottom) and normalized distribution of average activity (left) for SPNs classified as shuffle behavior silent (green), behavior-silent (activation occurrence below 2.5%), and non-silent cells (grey).

c, Average fraction of dSPNs (red; n = 33 sessions in 8 mice) and iSPNs (blue; n = 40 sessions in 9 mice) labeled shuffle behavior-silent for each behavior (left panel) and average fraction of dSPNs and iSPNs labeled shuffle behavior-silent during no behavior or one to five behaviors (right panel).

d, Simple matching coefficient for separating each behavior from other behaviors using neurons that are classified as either shuffle behavior-silent during proper behavior (hatched bars) or non-silent (unfilled bars) in dSPNs (red; n = 20 sessions in 8 mice) or iSPN recordings (blue; n = 25 sessions from 9 mice) (linear mixed effect model followed by post-hoc permutation-based two-sided t-test, behavior-inactive vs. non-behavior-inactive neurons: ns $p > 0.05$, * $p < 0.05$, ** $p < 0.01$, *** $p < 0.001$). The label "no data" for certain behaviors corresponds to instances for which not enough neurons were classified as shuffle behavior-silent to compute predictions in all sessions in all mice. For D2-SPNs, the results are similar to those obtained when the neurons are classified as behavior-silent and non-behavior-silent.

Abbreviation: loco., locomotion.

Data are presented as mean values ± SEM. Detailed statistics are displayed in Supplementary Table 1. Source data are provided as a Source Data file.

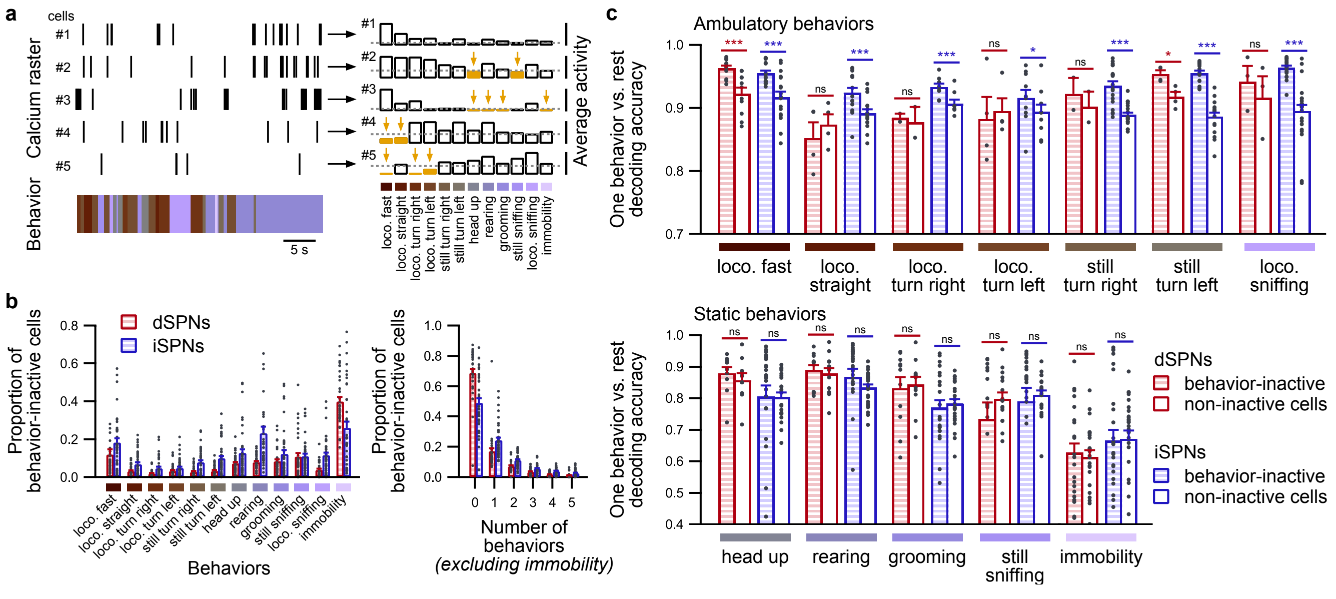

Supplementary Figure 15: Contribution of behavior-inactive cells to neural code using an alternate definition.

a, Behavior-inactive cells are identified according to their average activity during each behavior, as illustrated by 5 representative neurons (left panel) displaying different average activities for each behavior (right panel). The threshold for identifying an inhibited cell during a given behavior is set when the cell displays an average activity of less than 0.1 events/s (right panel, yellow bars).

b, Average fraction of dSPNs (red; n = 33 sessions in 8 mice) and iSPNs (blue; n = 40 sessions in 9 mice) labeled behavior-inactive for each behavior (left panel) and average fraction of dSPNs and iSPNs labeled behavior-inactive during no behavior or one to five behaviors (right panel).

c, Simple matching coefficient for separating each behavior from other behaviors using neurons that are classified as either behavior-inactive during proper behavior (hatched bars) or non-behavior-inactive (unfilled bars) in dSPNs (red; n = 29 sessions in 8 mice) or iSPN recordings (blue; n = 37 sessions from 9 mice) (linear mixed effect model followed by post-hoc permutation-based two-sided t-test, behavior-inactive vs. non-behavior-inactive neurons: ns $p > 0.05$, * $p < 0.05$, ** $p < 0.01$, *** $p < 0.001$). The results are similar to those obtained when the neurons are classified as behavior-silent and non-behavior-silent.

Abbreviation: loco., locomotion

Data are presented as mean values ± SEM. Detailed statistics are displayed in Supplementary Table 1. Source data are provided as a Source Data file.

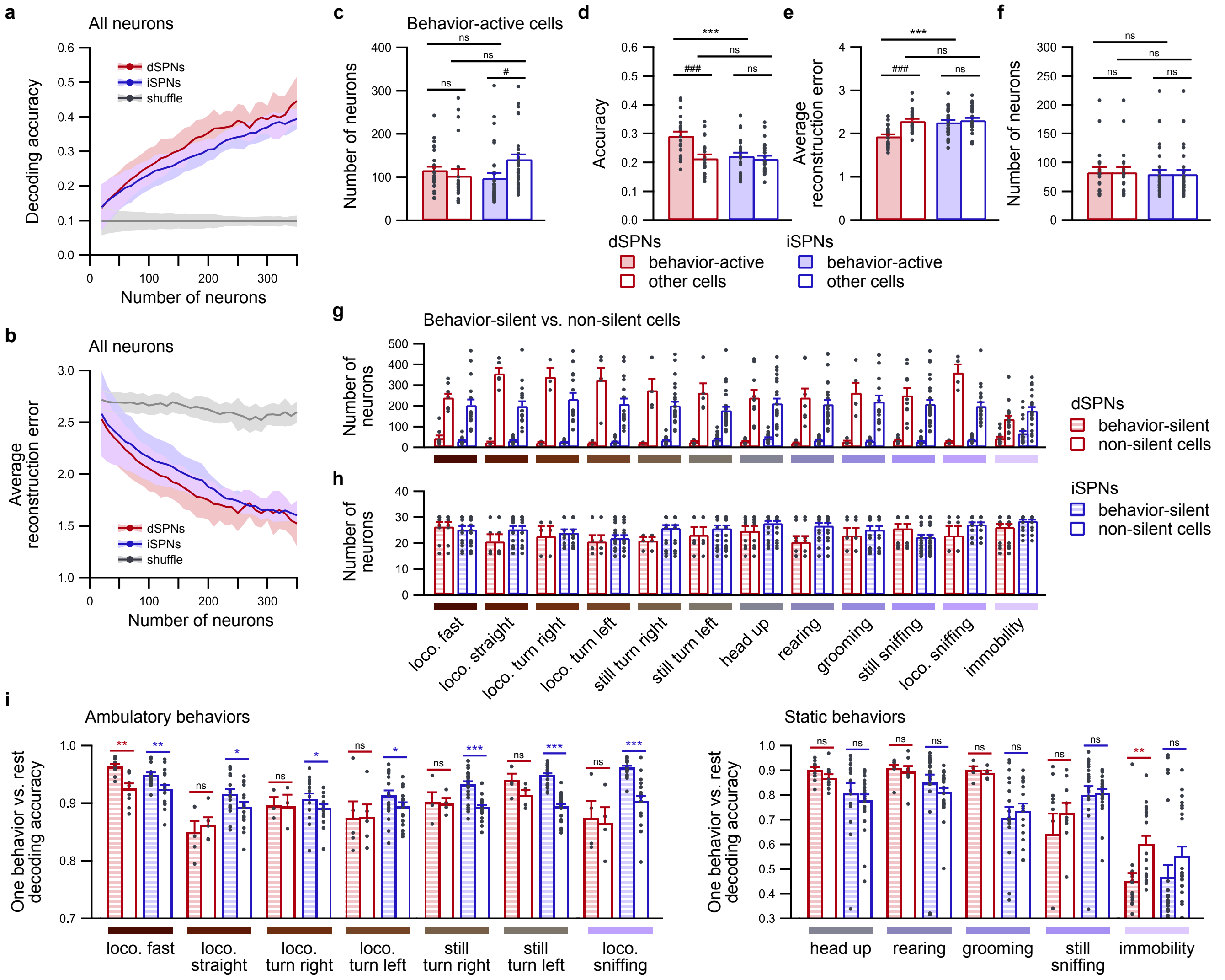

Supplementary Figure 16: Dependence of decoding performance on the number of cells used.

a-b, Relationship between the number of cells used and the behavior prediction accuracy (**a**) or the behavior reconstruction error (**b**).

c, For the prediction of behaviors using behavior-active of non-active cells, an uneven number of neurons between conditions was used (dSPNs: red, n = 29 sessions in 8 mice; iSPNs: blue, n = 37 sessions in 9 mice) (linear mixed effect model followed by post-hoc permutation-based two-sided t-test, dSPNs vs. iSPNs: ns $p > 0.05$; behavior-active vs. other cells: ns $p > 0.05$, # $p < 0.05$).

d-f, Decoding accuracy (**d**) and average reconstruction error (**e**) of SVM classifiers applied to behavior-active (plain bars) or non-behavior-active (unfilled bars) dSPNs (red; n = 29 sessions in 8 mice) or iSPNs (blue; n = 37 sessions in 9 mice) when an even number of neurons is used in all conditions (**f**) (linear mixed effect model followed by post-hoc permutation-based two-sided t-test, dSPNs vs. iSPNs: ns $p > 0.05$, *** $p < 0.01$; behavior-active vs. other cells: # $p < 0.05$). The results are similar to those obtained without controlling for the number of cells.

g, For the prediction of behaviors using behavior-silent of non-silent cells, an uneven number of neurons between conditions was used (dSPNs: red, n = 29 sessions in 8 mice; iSPNs: blue, n = 37 sessions in 9 mice).

h-i, Using a similar number of neurons in all conditions (**h**), simple matching coefficient for separating each behavior from other behaviors (**i**) using neurons that are classified as either behavior-silent during proper behavior (hatched bars) or non-behavior-silent (unfilled bars) in dSPNs (red; n = 29 sessions in 8 mice) or iSPN recordings (blue; n = 37 sessions from 9 mice) and (linear mixed effect model followed by post-hoc permutation-based two-sided t-test, behavior-inactive vs. non-behavior-inactive neurons: ns $p > 0.05$, * $p < 0.05$, ** $p < 0.01$, *** $p < 0.001$). The results are similar to those obtained without controlling for the number of cells.

Abbreviation: loco., locomotion

Data are presented as mean values ± SEM. Detailed statistics are displayed in Supplementary Table 1. Source data are provided as a Source Data file.

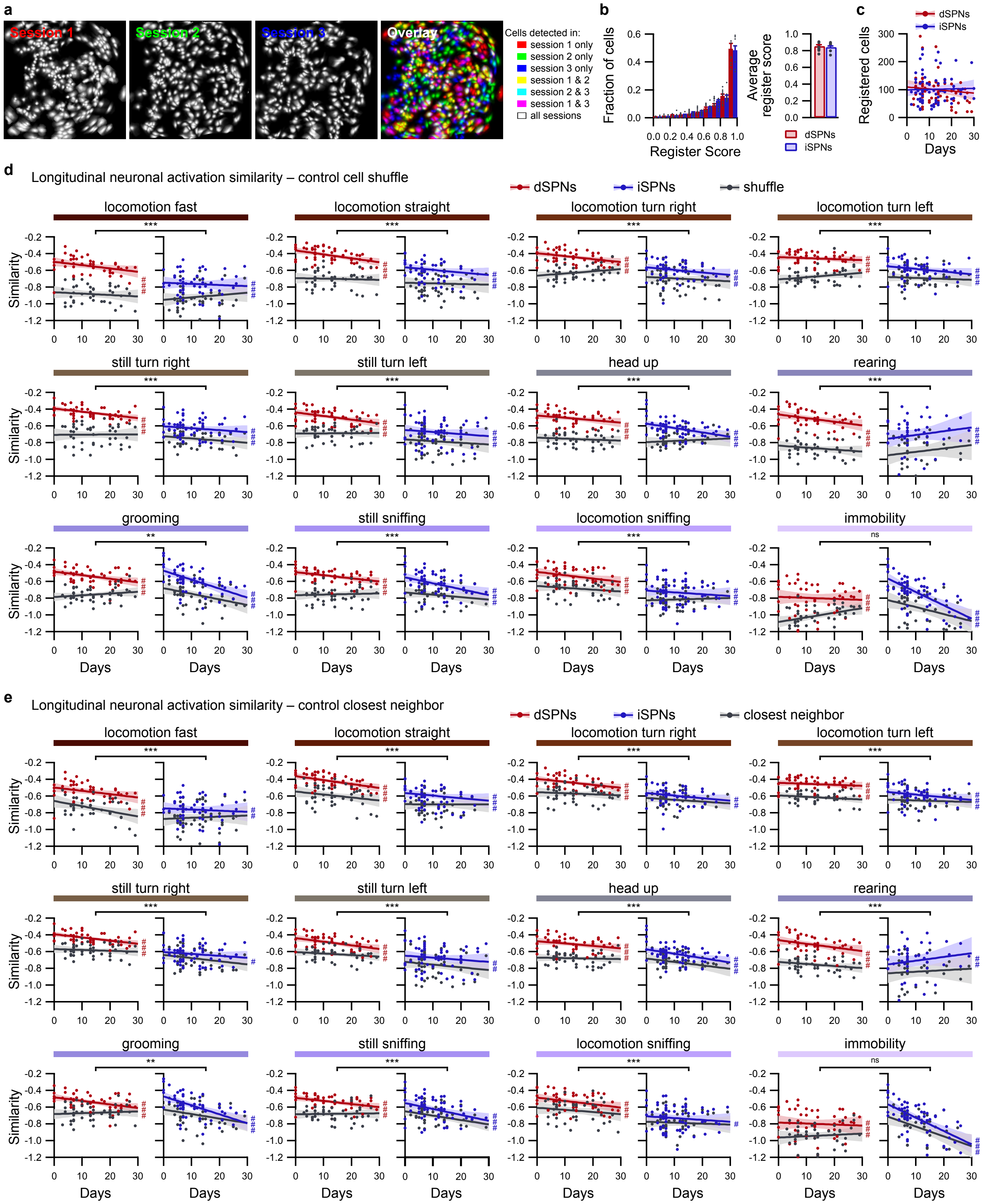

Supplementary Figure 17: Validation of longitudinal registration of cells and longitudinal evaluation of neuronal activation similarity during behaviors.

a, Representative example of spatial footprints of neurons detected during three different sessions recorded from the same mouse and an overlay of the aligned spatial footprint maps, which are color-coded according to the sessions during which the cells were detected.

b, Distribution of register scores between sessions (left panel) and mean register score (right panel) averaged for all D1 (red; n = 8) and D2 A2A (blue; n = 9) mice.
c, Number of registered cells between pairs of sessions from the same mouse as a function of the number of days between sessions for dSPN (red; n = 72 pairs of sessions in 8 mice) and iSPN recordings (blue; n = 80 sessions in 9 mice).
d-e, Evolution of the neuronal activation similarity during identified behaviors across days for dSPNs (red; n = 52 pairs of sessions in 8 mice) and iSPNs (blue; n = 62 pairs of sessions in 9 mice) compared to their respective controls (gray), which were obtained by shuffling pairs of registered neurons (**d**) or by replacing one neuron in each pair of registered cells by its spatially closest neighbor (**e**) (linear mixed effect model followed by post-hoc analysis of covariance, dSPNs vs. iSPNs: ns $p > 0.05$, ** $p < 0.01$, *** $p < 0.001$; observed data vs. control: $^{\#}$ $p < 0.05$, $^{\#\#}$ $p < 0.01$, $^{\#\#\#}$ $p < 0.001$).
Data are presented as mean values ± SEM. Detailed statistics are displayed in Supplementary Table 1. Source data are provided as a Source Data file.

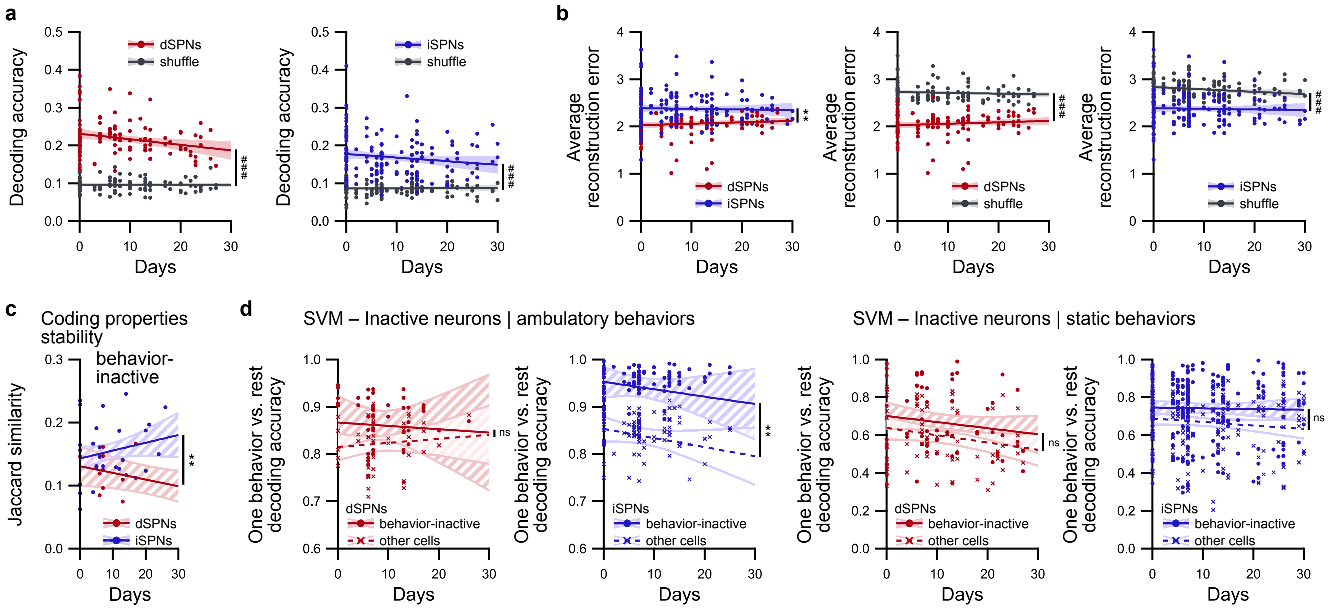

Supplementary Figure 18: Complements related to longitudinal prediction of behaviors.
a, Accuracy in predicting the behavior according to the activity of longitudinally registered neurons using SVM classifiers trained on a different recordings for dSPNs (red; n = 121 pairs of sessions) and iSPNs (blue; n = 171 pairs of sessions) and their respective controls, which were obtained using classifiers trained on time-lagged data (gray) (linear mixed effect model followed by post-hoc analysis of covariance, observed data vs. time-lagged: ### $p < 0.001$).
b, Average behavioral reconstruction error in predicting the behavior according to the activity of longitudinally registered neurons using SVM classifiers trained on different recordings for dSPNs (red; n = 121 pairs of sessions) and iSPNs (blue; n = 171 pairs of sessions) (linear mixed effect model followed by post-hoc analysis of covariance, dSPNs vs. iSPNs: ** $p < 0.01$; observed data vs. time-lagged: ### $p < 0.001$).
c, Quantification of the long-term stability of the coding properties of neurons using the Jaccard similarity coefficient between the binary classification of longitudinally registered labeled behavior-inactive in dSPN (red; n = 16 pairs of sessions meeting criterion) and iSPN recordings (blue; n = 29 pairs of sessions meeting criterion) (linear mixed effect model followed by post-hoc analysis of covariance, dSPNs vs. iSPNs: ** $p < 0.01$).
d, Simple matching coefficient for the long-term prediction of separating one behavior from other behaviors using neurons that were classified as behavior-inactive (circles, plain line, colored confidence interval) or non-behavior-inactive (crosses, dashed line, unfilled confidence interval) during this behavior, pooled for ambulatory behaviors (top panels) or static behaviors (bottom panels) for dSPNs (red; ambulatory: n = 20 pairs of sessions meeting criterion; static: n = 67 pairs of sessions meeting criterion) and iSPNs (blue; ambulatory: n = 46 pairs of sessions meeting criterion; static: n = 75 pairs of sessions meeting criterion) (linear mixed effect model followed by post-hoc analysis of covariance, behavior-inactive vs. non-behavior-inactive: ns $p > 0.05$, ** $p < 0.01$). Note that the results obtained for behavior-inactive neurons are highly similar to those observed for behavior-silent neurons.
Data are presented as mean values ± SEM. Detailed statistics are displayed in Supplementary Table 1. Source data are provided as a Source Data file.

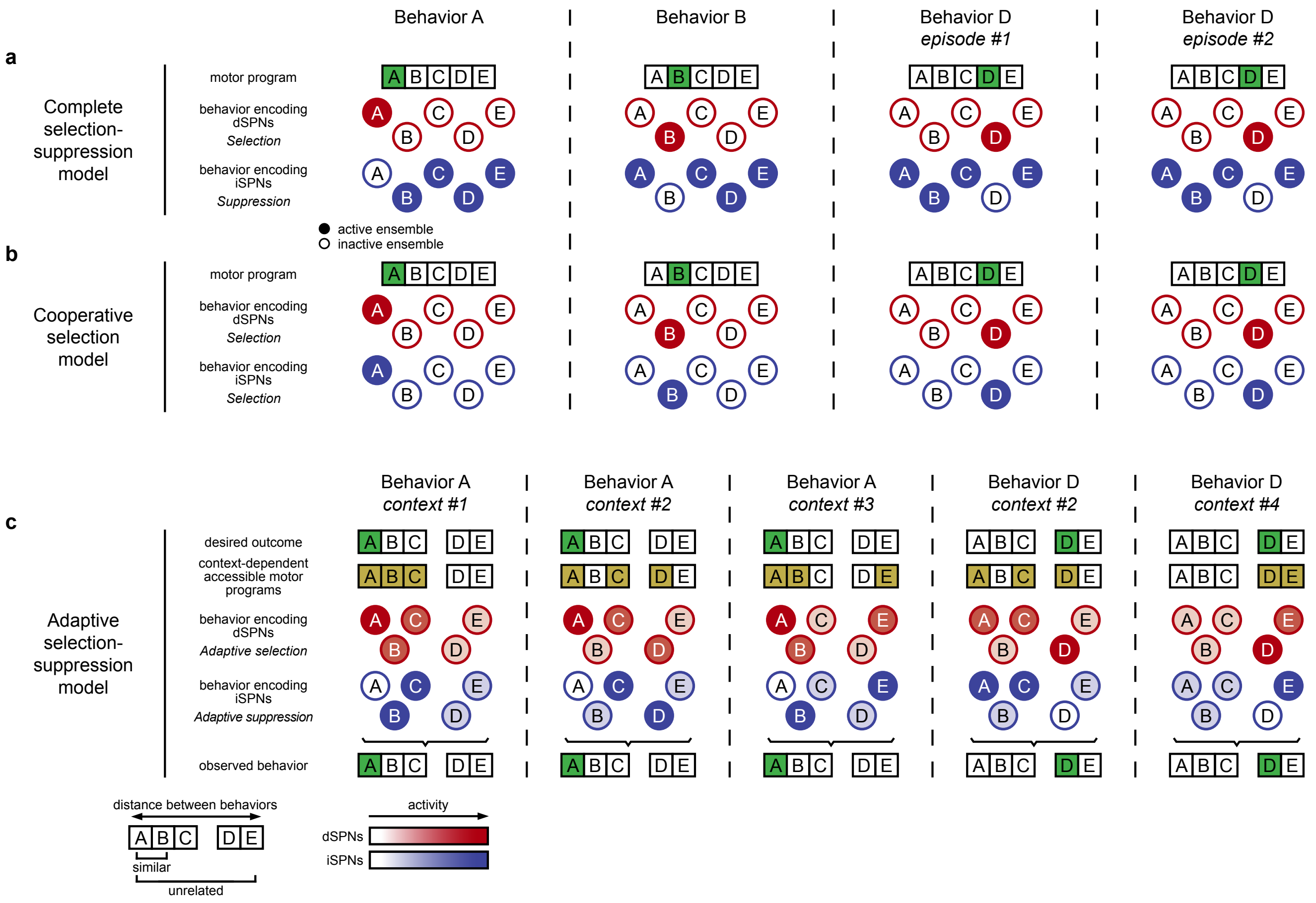

d

Adequacy between models and experimental observations

Experimental observations	Models: Complete selection-suppression model	Cooperative selection model	Adaptive selection-suppression model
Opposite pro- vs. antikinetic function of dSPNs and iSPNs	✓	✗	✓
Simlar sizes of dSPNs and iSPNs ensembles activated during actions	✗	✓	✓
Coactivations of subsets of dSPNs and iSPNs during actions	✗	✓	✓
Different patterns of neuronal activation for different occurrences of the same action	✗	✗	✓
Higher neuronal activation similarity in dSPNs than iSPNs	✗	✗	✓
dSPNs neural code biased toward activation	✓	✓	✓
iSPNs neural code biased toward silencing	✓	✗	✓

Supplementary Figure 19: Comparison of theoretical models describing SPN encoding of behaviors.

Multiple models have been formulated to describe the organization of the basal ganglia and capture the properties of striatal neurons.

a, In the "complete selection-suppression" model[1], subsets of dSPNs and iSPNs are concurrently activated during behaviors. While a specific subpopulation of dSPNs is activated to promote a given motor program, all iSPN subpopulations associated with other motor programs are active to simultaneously suppress these competing motor programs. This model predicts the widespread activation of iSPNs and the focused activation of specific dSPNs.

b, Alternatively, some models hypothesized a cooperative selection function for dSPNs and iSPNs based on concurrent coordinated activation in both pathways to select proper behaviors[2]. In this model, comparable populations of dSPNs and iSPNs are tuned toward specific behaviors and activated simultaneously to select proper motor programs. This model predicts a high and equivalent level of neuronal activation similarity in dSPNs and iSPNs during episodes of the

same behavior, as well as comparable neuronal activation similarities between behaviors in both SPN pathways.

c, In this study, we propose a new model, referred to here as the "adaptive selection suppression" model. We postulate that at any time, only a few reachable behaviors in the overall behavioral repertoire actual compete with the ongoing/most-desired behavior. These competing behaviors are highly dependent on the ongoing external context (and potentially dependent on the internal state of the animal) and thus differ between different episodes of a given behavior. We hypothesize that, as a result, dSPNs that encode the ongoing behavior and competing behaviors are activated, while dSPNs that encode other behaviors remain silent. At the same time, in the indirect pathway, iSPNs specifically associated with competing behaviors are activated to suppress these competing behaviors, while iSPNs associated with the ongoing behavior remain silent. As a consequence, the activations in the direct and indirect pathways, which select and suppress motor programs, respectively, result in the proper selection of only one ongoing motor program.

d, When assessing the adequacy between model predictions and experimental observations, we trust that the adaptive selection-suppression model captures more SPN properties, as established in previous studies. First, despite its accurate account of the antagonistic function of dSPNs and iSPNs[3-9], which implies a bias in the neural code toward activation and inhibition, respectively, the complete selection-suppression model proposes the widespread activation of iSPNs and focused activation of dSPNs during actions, which is not supported by recordings of neuronal activity[2,10-14]. Additionally, this model cannot explain the differences in neuronal activation similarity we observed in the present study, as this model predicts that populations of neurons that are activated during episodes of the same behaviors are highly similar for dSPNs and iSPNs and almost identical when comparing dSPNs and iSPNs. Furthermore, the cooperative selection model properly captures the simultaneous and targeted activation of subsets of dSPNs and iSPNs during actions[2,11,12]. However, this model does not explain the antagonistic functions of dSPNs and iSPNs, and because it proposes similar neuronal activation patterns for both dSPNs and iSPNs, it does not account for the differences in the tuning properties in the two pathways we observed in this study. Our proposed novel adaptive selection-suppression formulation aims to reconcile some of the above discrepancies. This model accounts for the prokinetic and antikinetic effects associated with activating dSPNs and iSPNs, respectively. This finding is supported by our observation that the neural code for behaviors is biased toward activation in dSPNs and silencing in iSPNs. Importantly, by incorporating the idea that neuronal activation patterns during self-paced spontaneous exploration of the behavioral repertoire are highly dependent on the current external and internal contexts, we propose that dSPNs and iSPNs activated during different occurrences of the same behavior differ. This result supports the context-dependent variability in activation patterns that we observed using the neuronal activation similarity measure. Moreover, the neuronal clusters in dSPNs that are associated with the ongoing behavior are consistently active, whereas there may be instances when, for the same observed behavior, the sets of activated iSPNs drastically differ (to inhibit different competing behaviors; see, for example, behavior A context #1 vs. behavior A context #3); thus, the proposed model predicts a higher neuronal activation similarity in dSPNs than in iSPNs. In addition, our model incorporates an important feature of the neural code, namely, that in response to the expressed motor program, specific subgroups of dSPNs are activated, whereas specific subgroups of iSPNs are consistently inactive.

Supplementary Table 1:

Detailed statistical analysis for Figures and Supplementary Figures.

FIGURE. NUMBER	BASIC DESCRIPTION	SAMPLE SIZE	TEST USED	RESULT	POST-HOC TEST	P-VALUE
Fig. 1b	Behaviors distribution in open-field	D1 mice:<br>33 sessions in 8 mice<br>A2A mice:<br>40 sessions in 9 mice	Linear mixed effect model followed by ANOVA (Statterthwaite degrees of freedom)	DF1 DF2 F stat p-val<br>(Intercept) 1 5075.7 2950.5 0<br>genotype 1 5075.7 5.7e-21 1<br>behaviors 11 25.974 20.572 4.4e-10<br>time 1 5075.7 8.5e-19 1<br>genotype:behaviors 11 25.975 1.441 0.214<br>genotype:time 1 5075.7 8.2e-19 1<br>behaviors:time 11 37.332 3.5882 1.6e-3<br>genotype:behaviors:time 11 37.332 0.559 0.847		
Fig. 1f<br>Supp. Fig. 4f	Quantification average per recording session	dSPNs:<br>33 sessions in 8 mice<br>iSPNs:<br>40 sessions in 9 mice	Permutation-based t-test	dSPNs vs. iSPNs: p-val<br>Number of cells 0.1012<br>Transient frequency 0.0062<br>Transient decay 0<br>Deconvolved activity 0		
Supp. Fig. 4g	Quantification average per mouse	D1:<br>8 mice<br>A2A:<br>9 mice	Permutation-based t-test	D1 vs. A2A: p-val<br>Number of cells 0.2204<br>Transient frequency 0.0688<br>Transient decay 0.0078<br>Deconvolved activity 0.0118		
Supp. Fig. 4h	Comparison A2A(Tg) vs. A2A(AAV) mice	A2A(Tg):<br>6 mice<br>A2A(AAV):<br>3 mice	Permutation-based t-test	A2A(Tg) vs. A2A(AAV): p-val<br>Number of cells 0.1076<br>Transient frequency 0.1094<br>Transient decay 0.0738<br>Deconvolved activity 0.11		
Fig.1g	Population activity per behavior – deconvolved activity	dSPNs:<br>33 sessions in 8 mice<br>iSPNs:<br>40 sessions in 9 mice	Linear mixed effect model followed by ANOVA (Statterthwaite degrees of freedom)	DF1 DF2 F stat p-val<br>(Intercept) 1 17.982 1208.6 6.1e-18<br>genotype 1 17.982 9.514 0.00064<br>behaviors 11 31.119 28.382 3.6e-13<br>genotype:behaviors 11 31.119 4.915 0.00022	Permutation-based t-test between genotypes for each behavior (corrected for multiple comparisons)	l. fast: 0.0904<br>l. str: 0.0044<br>l. right: 0<br>l. left: 0.0008<br>st. right: 0<br>st. left: 0.0022<br>head up: 0.143<br>rear.: 0.0008<br>groom.: 0.0246<br>st. sniff.: 0.3204<br>l. sniff.: 0.015<br>immo.: 0.0186
Supp. Fig. 4i	Population activity per behavior – raw calcium activity	dSPNs:<br>33 sessions in 8 mice<br>iSPNs:<br>40 sessions in 9 mice	Linear mixed effect model followed by ANOVA (Statterthwaite degrees of freedom)	DF1 DF2 F stat p-val<br>(Intercept) 1 15.902 15.10 0.0013<br>genotype 1 15.902 0.356 0.559<br>behaviors 11 35.421 3.255 1.6e-7<br>genotype:behaviors 11 35.421 2.369 0.026	Permutation-based t-test between genotypes for each behavior (corrected for multiple comparisons)	l. fast: 0.113<br>l. str: 0.035<br>l. right: 0.0018<br>l. left: 0.048<br>st. right: 0.0002<br>st. left: 0.1818<br>head up: 0.0112<br>rear.: 0.354<br>groom.: 0.247<br>st. sniff.: 0.0002<br>l. sniff.: 0.313<br>immo.: 0
Supp. Fig. 4j	Population activity per behavior – calcium transients	dSPNs:<br>33 sessions in 8 mice<br>iSPNs:<br>40 sessions in 9 mice	Linear mixed effect model followed by ANOVA (Statterthwaite degrees of freedom)	DF1 DF2 F stat p-val<br>(Intercept) 1 16.908 238.2 2.1e-11<br>genotype 1 16.908 3.456 0.0793<br>behaviors 11 45.961 2.387 0.0196<br>genotype:behaviors 11 34.961 0.115 0.9998	Permutation-based t-test between genotypes for each behavior (corrected for multiple comparisons)	l. fast: 0.176<br>l. str: 0.0914<br>l. right: 0.246<br>l. left: 0.0498<br>st. right: 0.076<br>st. left: 0.221<br>head up: 0.221<br>rear.: 0.334<br>groom.: 0.113<br>st. sniff.: 0.064<br>l. sniff.: 0.117<br>immo.: 0.287
Fig.2b<br>Supp. Fig. 5a-b	Neuronal activation similarity	dSPNs:<br>33 sessions in 8 mice<br>iSPNs:<br>40 sessions in 9 mice	Linear mixed effect model followed by ANOVA (Statterthwaite degrees of freedom)	DF1 DF2 F p-val<br>(Intercept) 1 17.908 648.4 1.6e-13<br>genotype 1 17.908 10.20 0.0051<br>behaviors 11 21.023 39.77 1.5e-11<br>shuffle 1 18.29 699.6 4.9e-16<br>genotype:behaviors 11 21.023 2.932 0.0156<br>genotype:shuffle 1 18.29 4.191 0.0453<br>behaviors:shuffle 11 35.769 6.107 1.6e-5<br>genotype:behaviors:shuffle 11 35.769 1.187 0.3304	Permutation-based t-test between genotypes for each behavior and paired t-test between observed data and shuffle (corrected for multiple comparisons)	dSPNs vs. iSPNs:<br>l. fast: 0.007<br>l. str: 0<br>l. right: 0<br>l. left: 0.202<br>st. right: 0<br>st. left: 0<br>head up: 0.017<br>rear.: 0.0006<br>groom.: 0.4352<br>st. sniff.: 0.144<br>l. sniff.: 0<br>immo.: 0.4512<br><br>for all comparisons vs. shuffle:<br>p = 0
Fig. 2c	Inverse coefficient of variation	dSPNs:<br>33 sessions in 8 mice<br>iSPNs:<br>40 sessions in 9 mice	Linear mixed effect model followed by ANOVA (Statterthwaite degrees of freedom)	DF1 DF2 F p-val<br>(Intercept) 1 18.001 2915 2.3e-21<br>genotype 1 18.001 11.57 0.0032<br>behaviors 11 27.941 24.83 1.5e-11<br>genotype:behaviors 11 27.941 3.437 0.0041	Permutation-based t-test between genotypes for each behavior (corrected for multiple comparisons)	l. fast: 0.0014<br>l. str: 0<br>l. right: 0<br>l. left: 0<br>st. right: 0<br>st. left: 0<br>head up: 0.0066<br>rear.: 0

						groom.: 0.0114<br>st. sniff.: 0.324<br>l. sniff.: 0<br>immo.: 0.0056
Supp. Fig. 5c	Neuronal activation similarity / temporal partitions	dSPNs:<br>33 sessions in 8 mice<br>iSPNs:<br>40 sessions in 9 mice	Linear mixed effect model followed by ANOVA (Statterthwaite degrees of freedom)	DF1 DF2 F p-val<br>(Intercept) 1 18.014 1840.1 1.3e-19<br>genotype 1 18.014 20.141 0.00028<br>behaviors 11 19.114 139.86 7.8e-18<br>partition 9 8144.2 18.567 5.4e-31<br>genotype:behaviors 11 19.114 4.248 0.0028<br>genotype:partition 9 8144.2 0.736 0.676<br>behaviors:partition 99 8144.2 0.275 1<br>genotype:behaviors:partition 99 8144.2 0.314 1	Permutation-based t-test between genotypes for each behavior for each partition (corrected for multiple comparisons)	dSPNs vs. iSPNs:<br>(ordered for behaviors l. fast | l. str | l. right | l. left | st. right | st. left | head up | rear. | groom. | st. sniff. | l. sniff. | immo)<br><br>partition 1: 0.007 Í 0 Í 0.0002 Í 0.0208 Í 0 Í 0 Í 0.0198 Í 0.0004 Í 0.4446 Í 0.154 Í 0 Í 0.4452<br>partition 2: 0.0254 Í 0.0006 Í 0 Í 0.002 Í 0 Í 0 Í 0.065 Í 0 Í 0.3094 Í 0.0378 Í 0 Í 0.3254<br>partition 3: 0.0066 Í 0 Í 0 Í 0.0026 Í 0 Í 0 Í 0.1438 Í 0.0004 Í 0.4696 Í 0.0696 Í 0 Í 0.2508<br>partition 4: 0.0028 Í 0.0002 Í 0.0004 Í 0.0016 Í 0 Í 0 Í 0.0432 Í 0 Í 0.0788 Í 0.117 Í 0 Í 0.4052<br>partition 5: 0.059 Í 0.0004 Í 0 Í 0.0112 Í 0 Í 0 Í 0.0858 Í 0.0122 Í 0.2742 Í 0.0968 Í 0 Í 0.4454<br>partition 6: 0.0042 Í 0.0002 Í 0.0004 Í 0.0044 Í 0 Í 0 Í 0.0962 Í 0.0002 Í 0.388 Í 0.0174 Í 0 Í 0.3058<br>partition 7: 0.0068 Í 0.0002 Í 0 Í 0.0108 Í 0 Í 0 Í 0.0346 Í 0.001 Í 0.3648 Í 0.045 Í 0 Í 0.3128<br>partition 8: 0.0108 Í 0 Í 0 Í 0.0004 Í 0 Í 0 Í 0.0374 Í 0.0002 Í 0.2602 Í 0.0406 Í 0 Í 0.1764<br>partition 9: 0.006 Í 0 Í 0 Í 0.0032 Í 0 Í 0 Í 0.0134 Í 0 Í 0.3644 Í 0.0244 Í 0 Í 0.475<br>partition 10: 0.0092 Í 0.0004 Í 0 Í 0.043 Í 0 Í 0 Í 0.1148 Í 0.004 Í 0.1824 Í 0.0226 Í 0 Í 0.49
Supp. Fig. 6a	Neuronal activation similarity odd vs. even	dSPNs:<br>33 sessions in 8 mice<br>iSPNs:<br>40 sessions in 9 mice	Linear mixed effect model followed by ANOVA (Statterthwaite degrees of freedom)	DF1 DF2 F p-val<br>(Intercept) 1 19.824 16635 1.8e-30<br>genotype 1 19.824 17.161 0.00051<br>behaviors 11 23.519 40.031 1.5e-12<br>genotype:behaviors 11 23.519 2.262 0.0467	Permutation-based t-test between genotypes for each behavior (corrected for multiple comparisons)	l. fast: 0.4634<br>l. str: 0<br>l. right: 0.0012<br>l. left: 0.0325<br>st. right: 0<br>st. left: 0<br>head up: 0.487<br>rear.: 0.0376<br>groom.: 0.02<br>st. sniff.: 0.483<br>l. sniff.: 0<br>immo.: 0.012
Supp. Fig. 6b	Neuronal activation similarity one every two bouts	dSPNs:<br>33 sessions in 8 mice<br>iSPNs:<br>40 sessions in 9 mice	Linear mixed effect model followed by ANOVA (Statterthwaite degrees of freedom)	DF1 DF2 F p-val<br>(Intercept) 1 18.022 595.5 2.9e-21<br>genotype 1 18.022 15.873 5.9e-5<br>behaviors 11 24.312 18.86 3.2e-13<br>genotype:behaviors 11 24.312 2.784 0.075	Permutation-based t-test between genotypes for each behavior (corrected for multiple comparisons)	l. fast: 0.0126<br>l. str: 0<br>l. right: 0<br>l. left: 0.0008<br>st. right: 0<br>st. left: 0<br>head up: 0.0218<br>rear.: 0<br>groom.: 0.297<br>st. sniff.: 0.0038<br>l. sniff.: 0<br>immo.: 0.4762
Supp. Fig. 6c	Neuronal activation similarity – Dot product	dSPNs:<br>33 sessions in 8 mice<br>iSPNs:<br>40 sessions in 9 mice	Linear mixed effect model followed by ANOVA (Statterthwaite degrees of freedom)	DF1 DF2 F p-val<br>(Intercept) 1 17.564 16707 1.3e-27<br>genotype 1 17.564 21.51 0.0002<br>behaviors 11 21.884 23.09 4.3e-10<br>genotype:behaviors 11 21.884 2.791 0.0173	Permutation-based t-test between genotypes for each behavior (corrected for multiple comparisons)	l. fast: 0.011<br>l. str: 0<br>l. right: 0<br>l. left: 0.0172<br>st. right: 0<br>st. left: 0<br>head up: 0.0138<br>rear.: 0.0006<br>groom.: 0.4428<br>st. sniff.: 0.092<br>l. sniff.: 0<br>immo.: 0.4062
Supp. Fig. 6d	Neuronal activation similarity – Raw calcium activity	dSPNs:<br>33 sessions in 8 mice<br>iSPNs:<br>40 sessions in 9 mice	Linear mixed effect model followed by ANOVA (Statterthwaite degrees of freedom)	DF1 DF2 F p-val<br>(Intercept) 1 17.968 458.0 3.1e-14<br>genotype 1 17.968 15.63 0.0009<br>behaviors 11 25.200 19.19 1.5e-9<br>shuffle 1 18.087 3.226 0.0843<br>genotype:behaviors 11 25.200 3.049 0.01<br>genotype:shuffle 1 18.087 9.375 0.00067<br>behaviors:shuffle 11 35.433 80.58 8.3e-7<br>genotype:behaviors:shuffle 11 35.433 0.7549 0.6802	Permutation-based t-test between genotypes for each behavior and paired t-test between observed data and shuffle (corrected for multiple comparisons)	dSPNs vs. iSPNs:<br>l. fast: 0.0116<br>l. str: 0.0046<br>l. right: 0<br>l. left: 0.0004<br>st. right: 0.0002<br>st. left: 0.0044<br>head up: 0.0594<br>rear.: 0<br>groom.: 0.0014<br>st. sniff.: 0<br>l. sniff.: 0.0006<br>immo.: 0<br><br>observed data vs.

						shuffle:<br>dSPNs | iSPNs<br>l. fast: 0.0002 | 0.0134<br>l. str: 0.0412 | 0.299<br>l. right: 0.014 | 0.04<br>l. left: 0.116 | 0.273<br>st. right: 0.496 | 0<br>st. left: 0.210 | 0<br>head up: 0.488 | 0.285<br>rear.: 0 | 0.0392<br>groom.: 0.0006 | 0.201<br>st. sniff.: 0.443| 0<br>l. sniff.: 0.2382 | 0.0004<br>immo.: 0.0222 | 0.090
Supp. Fig. 7b	Average behaviors distribution – effect of saline or amphetamine	Salin<br>D1 mice:<br>12 sessions in 8 mice<br>A2A mice:<br>11 sessions in 9 mice<br>Amphet<br>D1 mice:<br>7 sessions in 7 mice<br>A2A mice:<br>8 sessions in 8 mice	Linear mixed effect model followed by ANOVA (Statterthwaite degrees of freedom)	DF1 DF2 F p-val<br>(Intercept) 1 739.8 11482 0<br>genotype 1 739.8 2.0e-17 1<br>behaviors 11 20.73 26.7 8.8e-10<br>drugs 1 739.8 9.8e-17 1<br>pre-post 1 739.8 1.6e-15 1<br>genotype:behaviors 11 20.73 1.690 0.146<br>genotype:drugs 1 739.8 1.2e-16 1<br>behaviors:drugs 11 24.61 9.501 2.2e-6<br>genotype:pre-post 1 739.8 9.3e-17 1<br>behaviors:pre-post 11 25.89 7.344 1.6e-5<br>drugs:pre-post 1 739.8 3.9e-15 1<br>geno:behav:drugs 11 24.61 1.482 0.201<br>geno:behav:pre-post 11 25.89 2.050 0.065<br>geno:drug:pre-post 1 739.8 1.0e-15 1<br>behav:drug:pre-post 11 25.80 17.410 3.0e-9<br>geno:behav:drug:pre-post 11 25.80 1.592 0.1602	Permutation-based t-test between pre- and post-injection periods for each behavior (pooled for both genotypes) (corrected for multiple comparisons)	Pre vs. post:<br>saline | amphet<br>l. fast: 0 | 0<br>l. str: 0 | 0.0004<br>l. right: 0 | 0.0038<br>l. left: 0 | 0<br>st. right: 0.0016 | 0.0148<br>st. left: 0.004 | 0.162<br>head up: 0.220 | 0.0020<br>rear.: 0.055 | 0<br>groom.: 0.001 | 0<br>st. sniff.: 0.237| 0.4868<br>l. sniff.: 0 | 0.0346<br>immo.: 0.011 | 0.1430
Supp. Fig. 7c	Population activity during baseline and post-injection periods	Salin<br>D1 mice:<br>12 sessions in 8 mice<br>A2A mice:<br>11 sessions in 9 mice<br>Amphet<br>D1 mice:<br>7 sessions in 7 mice<br>A2A mice:<br>8 sessions in 8 mice	Linear mixed effect model followed by ANOVA (Statterthwaite degrees of freedom)	DF1 DF2 F p-val<br>(Intercept) 1 17.92 808.9 2.3e-16<br>genotype 1 17.92 9.936 0.0055<br>drug 1 16.51 16.26 9.1e-4<br>pre-post 1 18.42 35.03 1.2e-5<br>genotype:drugs 1 16.51 2.334 0.145<br>genotype:pre-post 1 18.42 2.883 0.106<br>drug:pre-post 1 15.76 0.021 0.888<br>geno:drug:pre-post 1 15.76 6.991 0.0178	Permutation-based t-test between pre- and post-injection periods and between treatments (corrected for multiple comparisons)	Pre vs. post<br>saline | amphet<br>dSPNs: 0 | 0.452<br>iSPNs: 0.0004 | 0<br><br>Salin vs amphet<br>dSPNs: 0.0428<br>iSPNs: 0.421
Supp. Fig. 7d	Population activity per behavior – effect of saline or amphetamine	Salin<br>dSPNs:<br>12 sessions in 8 mice<br>iSPNs:<br>11 sessions in 9 mice<br>Amphet<br>dSPNs:<br>7 sessions in 7 mice<br>iSPNs:<br>8 sessions in 8 mice	Linear mixed effect model followed by ANOVA (Statterthwaite degrees of freedom)	DF1 DF2 F p-val<br>(Intercept) 1 17.498 1300.5 7.2e-18<br>genotype 1 17.498 13.639 0.0017<br>behaviors 11 17.486 37.942 5.9e-10<br>drugs 1 7.633 102.17 1.1e-5<br>pre-post 1 17.214 84.965 5.5e-8<br>genotype:behaviors 11 17.486 8.580 5.0e-5<br>genotype:drugs 1 7.633 0.9599 0.357<br>behaviors:drugs 11 16.729 4.536 0.0029<br>genotype:pre-post 1 17.214 11.047 0.004<br>behaviors:pre-post 11 19.653 3.196 0.012<br>drugs:pre-post 1 15.736 14.394 0.0016<br>geno:behav:drugs 11 16.729 1.872 0.120<br>geno:behav:pre-post 11 19.653 1.331 0.279<br>geno:drug:pre-post 1 15.736 13.334 0.0022<br>behav:drug:pre-post 11 19.676 1.429 0.236<br>geno:behav:drug:pre-post 11 19.676 1.834 0.1161	Permutation-based t-test between pre- and post-injection periods for each genotype and between treatments for each condition (corrected for multiple comparisons)	Pre vs. post:<br>dSPNs<br>saline | amphet<br>l. fast: 0.248 | 0.0128<br>l. str: 0.258 | 0.0286<br>l. right: 0.115 | 0.0354<br>l. left: 0.412 | 0.0018<br>st. right: 0.0634 | 0.292<br>st. left: 0.096 | 0.127<br>head up: 0.128 | 0.368<br>rear.: 0.212 | 0.243<br>groom.: 0.124 | 0.141<br>st. sniff.: 0.211 | 0.235<br>l. sniff.: 0.231 | 0.046<br>immo.: 0.386 | 0.254<br><br>Pre vs. post:<br>iSPNs<br>saline | amphet<br>l. fast: 0.0358 | 0<br>l. str: 0.195 | 0<br>l. right: 0.146 | 0.0002<br>l. left: 0.0742 | 0<br>st. right: 0.123 | 0.0002<br>st. left: 0.326 | 0.0012<br>head up: 0.434 | 0.069<br>rear.: 0.336 | 0.0474<br>groom.: 0.451 | 0.005<br>st. sniff.: 0.243 | 0.0008<br>l. sniff.: 0.0304 | 0<br>immo.: 0.345 | 0.004<br><br>saline vs. amphet<br>baseline<br>dSPNs | iSPNs<br>l. fast: 0.105 | 0.0548<br>l. str: 0.112 | 0.222<br>l. right: 0.0832 | 0.397<br>l. left: 0.0646 | 0.270<br>st. right: 0.0745 | 0.488<br>st. left: 0.159 | 0.365<br>head up: 0.271 | 0.192<br>rear.: 0.382 | 0.470<br>groom.: 0.151 | 0.497<br>st. sniff.: 0.162 | 0.190<br>l. sniff.: 0.108 | 0.433<br>immo.: 0.418 | 0.435<br><br>saline vs. amphet<br>post-injection<br>dSPNs | iSPNs<br>l. fast: 0.0054 | 0.001<br>l. str: 0.0008 | 0<br>l. right: 0.003 | 0.0018<br>l. left: 0 | 0.0002<br>st. right: 0.207 | 0.0038<br>st. left: 0.0188 | 0.0014<br>head up: 0.245 | 0.0952<br>rear.: 0.376 | 0.0606<br>groom.: 0.119 | 0.0002

<table>
<tr><td></td><td></td><td></td><td></td><td></td><td></td><td>st. sniff.: 0.424 | 0.0128
l. sniff.: 0.0006 | 0
immo.: 0.378 | 0.0074</td></tr>
<tr><td>Supp. Fig. 8b</td><td>Neuronal activation similarity – effect of amphetamine</td><td>Salin
dSPNs:
12 sessions in 8 mice
iSPNs:
11 sessions in 9 mice
Amphet
iSPNs:
7 sessions in 7 mice
iSPNs:
8 sessions in 8 mice</td><td>Linear mixed effect model followed by ANOVA (Statterthwaite degrees of freedom)</td><td>DF1 DF2 F p-val
(Intercept) 1 13.76 526.5 2.3e-12
genotype 1 13.76 1.1461 0.0247
behaviors 11 14.267 15.747 4.2e-6
drug 1 15.701 124.49 7.2e-9
genotype:behaviors 11 14.267 2.5 0.0535
genotype:drug 1 15.701 2.310 0.148
behaviors:drug 11 16.998 7.196 0.00018
genotype:behaviors:drug 11 16.998 3.976 0.0295</td><td>Permutation-based t-test between genotypes for each behavior and between saline and amphetamine for each genotype and behavior
(corrected for multiple comparisons)</td><td>dSPNs vs. iSPNs:
saline | amphet
l. fast: 0.0072 | 0.469
l. str: 0.0084 | 0.4116
l. right: 0.0108 | 0.247
l. left: 0.1078 | 0.456
st. right: 0.0012 | 0.227
st. left: 0.0046 | 0.073
head up: 0.162 | 0.0932
rear.: 0.0214 | 0.3764
groom.: 0.0852 | 0.3116
st. sniff.: 0.069 | 0.299
l. sniff.: 0.0614 | 0.0046
immo.: 0.272 | 0.4376

saline vs. amphet
dSPNs | iSPNs
l. fast: 0.011 | 0.499
l. str: 0 | 0.004
l. right: 0 | 0.0196
l. left: 0.0002 | 0.0216
st. right: 0 | 0.0178
st. left: 0 | 0.021
head up: 0 | 0.0002
rear.: 0 | 0.0822
groom.: 0 | 0.0002
st. sniff.: 0 | 0.009
l. sniff.: 0.005 | 0.0114
immo.: 0.0028 | 0.0112</td></tr>
<tr><td>Fig. 3f</td><td>Correlation neuronal similarity vs. behavioral similarity</td><td>dSPNs:
33 sessions in 8 mice
iSPNs:
40 sessions in 9 mice</td><td>Permutation-based t-test</td><td>dSPNs vs. iSPNs: p = 0.0005</td><td></td><td></td></tr>
<tr><td>Supp. Fig. 9a</td><td>Correlation neuronal similarity vs. behavioral similarity – control similarity measure</td><td>dSPNs:
33 sessions in 8 mice
iSPNs:
40 sessions in 9 mice</td><td>Permutation-based t-test</td><td>dSPNs vs. iSPNs: p = 0.0026</td><td></td><td></td></tr>
<tr><td>Supp. Fig. 9b</td><td>Correlation neuronal similarity vs. behavioral similarity – 10 min</td><td>dSPNs:
24 sessions in 8 mice
iSPNs:
21 sessions in 9 mice</td><td>Permutation-based t-test</td><td>dSPNs vs. iSPNs: p = 0.2236</td><td></td><td></td></tr>
<tr><td>Supp. Fig. 9c</td><td>Correlation neuronal similarity vs. behavioral similarity – effect of duration analyzed</td><td>dSPNs:
33 sessions in 8 mice
iSPNs:
40 sessions in 9 mice</td><td>Linear mixed effect model followed by ANOVA (Statterthwaite degrees of freedom)</td><td>DF1 DF2 F p-val
(Intercept) 1 345 1125.1 1e-110
genotype 1 345 0.71495 0.398
duration 1 345 60.965 7.0e-14
genotype:duration 1 345 2.3176 0.1288</td><td>Permutation-based t-test between genotypes for each duration
(corrected for multiple comparisons)</td><td>dSPNs vs. iSPNs:
0–5 min: 0.1348
0–10 min: 0.3518
0–15 min: 0.0244
0–20 min: 0.0022
0–25 min: 0.0014
0–30 min: 0.0005</td></tr>
<tr><td>Supp. Fig. 9d</td><td>Neuronal activation similarity – 10 min</td><td>dSPNs:
24 sessions in 8 mice
iSPNs:
21 sessions in 9 mice</td><td>Linear mixed effect model followed by ANOVA (Statterthwaite degrees of freedom)</td><td>DF1 DF2 F p-val
(Intercept) 1 17.968 595.49 3.2e-15
genotype 1 17.968 15.873 0.00087
behaviors 11 30.166 18.86 1.4e-10
genotype:behaviors 11 30.165 2.7836 0.0127</td><td>Permutation-based t-test between genotypes for each behavior
(corrected for multiple comparisons)</td><td>l. fast: 0.0932
l. str: 0.0006
l. right: 0
l. left: 0.0008
st. right: 0
st. left: 0
head up: 0.0772
rear.: 0.002
groom.: 0.0664
st. sniff.: 0.481
l. sniff.: 0
immo.: 0.4084</td></tr>
<tr><td>Fig. 4b</td><td>SVM prediction – accuracy</td><td>dSPNs:
31 sessions in 8 mice
iSPNs:
38 sessions in 9 mice</td><td>Linear mixed effect model followed by ANOVA (Statterthwaite degrees of freedom)</td><td>DF1 DF2 F p-val
(Intercept) 1 18 311.76 4.8e-13
genotype 1 18 0.0755 0.787
shuffle 1 17.998 180.49 8.0e-11
genotype:shuffle 1 17.998 0.0166 0.899</td><td>Permutation-based t-test between genotypes for and paired t-test between observed data and shuffle
(corrected for multiple comparisons)</td><td>dSPNs vs. iSPNs: 0.292

observed vs. shuffle:
dSPNs: 0
iSPNs: 0</td></tr>
<tr><td>Fig. 4c</td><td>SVM prediction – error</td><td>dSPNs:
31 sessions in 8 mice
iSPNs:
38 sessions in 9 mice</td><td>Linear mixed effect model followed by ANOVA (Statterthwaite degrees of freedom)</td><td>DF1 DF2 F p-val
(Intercept) 1 17.977 1525.1 7.8e-19
genotype 1 17.977 0.102 0.753
shuffle 1 17.859 372.84 2.1e-13
genotype:shuffle 1 17.859 1.0101 0.328</td><td>Permutation-based t-test between genotypes for and paired t-test between observed data and shuffle
(corrected for multiple comparisons)</td><td>dSPNs vs. iSPNs: 0.137

observed vs. shuffle:
dSPNs: 0
iSPNs: 0</td></tr>
<tr><td>Fig. 4d</td><td>SVM accuracy per learner</td><td>dSPNs:
31 sessions in 8 mice
iSPNs:
38 sessions in 9 mice</td><td>Linear mixed effect model followed by ANOVA (Statterthwaite degrees of freedom)</td><td>DF1 DF2 F p-val
(Intercept) 1 17.844 2113.6 5.5e-20
genotype 1 17.844 0.399 0.536
distance 1 17.924 54.949 7.3e-7
shuffle 1 17.874 37.095 9.7e-6
genotype:distance 1 17.924 1.619 0.219
genotype:shuffle 1 17.874 1.441 0.246
distance:shuffle 1 17.664 55.09 7.9e-7
genotype:distance:shuffle 1 17.664 2.21 0.155</td><td></td><td></td></tr>
<tr><td>Supp. Fig. 16a</td><td>SVM prediction accuracy – dependency to the number of cells used</td><td>dSPNs:
31 sessions in 8 mice
iSPNs:
38 sessions in 9 mice</td><td>Linear mixed effect model followed by ANOVA (Statterthwaite degrees of freedom)</td><td>DF1 DF2 F p-val
(Intercept) 1 17.724 67225 4.9e-42
nb_cells 1 17.035 240.0 1.8e-11
genotype 1 17.724 4.782 0.0653
shuffle 1 18.107 42924 4.6e-41
nb_cells: genotype 1 17.035 0.3553 0.559
nb_cells:shuffle 1 16.282 311.7 3.0e-12</td><td></td><td></td></tr>
</table>

				genotype:shuffle 1 18.107 2.418 0.137<br>nb_cells:genotype:shuffle 1 16.282 17.11 0.0008		
Supp. Fig. 16b	SVM prediction accuracy – dependency to the number of cells used	dSPNs: 31 sessions in 8 mice<br>iSPNs: 38 sessions in 9 mice	Linear mixed effect model followed by ANOVA (Statterthwaite degrees of freedom)	DF1 DF2 F p-val<br>(Intercept) 1 18.106 5013.7 1.4e-23<br>nb_cells 1 17.858 190.0 5.9e-11<br>genotype 1 18.106 0.3643 0.554<br>shuffle 1 17.879 9.760 0.0059<br>nb_cells: genotype 1 17.858 2.998 0.101<br>nb_cells:shuffle 1 17.345 174.0 1.7e-10<br>genotype:shuffle 1 17.879 0.6923 0.416<br>nb_cells:genotype:shuffle 1 17.345 2.905 0.106		
Supp. Fig. 8c	SVM prediction accuracy – effect of amphetamine	Salin<br>dSPNs: 10 sessions in 6 mice<br>iSPNs: 13 sessions in 9 mice<br>Amphet<br>dSPNs: 6 sessions in 6 mice<br>iSPNs: 9 sessions in 9 mice	Linear mixed effect model followed by ANOVA (Statterthwaite degrees of freedom)	DF1 DF2 F p-val<br>(Intercept) 1 18.113 974 3.4e-17<br>genotype 1 18.113 1.705 0.208<br>drug 1 17.641 23.804 0.00013<br>shuffle 1 18.635 114.8 2.1e-9<br>genotype:drug 1 17.641 1.532 0.232<br>genotype:shuffle 1 18.635 0.438 0.516<br>drug:shuffle 1 18.536 30.03 3.0e-5<br>genotype:drug:shuffle 1 18.536 1.802 0.196	Permutation-based t-test between genotypes for and paired t-test between observed data and shuffle (corrected for multiple comparisons)	dSPNs vs. iSPNs:<br>salin: 0.4632<br>amphet: 0.0174<br><br>salin vs. amphet<br>dSPNs: 0.0226<br>iSPNs: 0<br><br>observed vs. shuffle<br>dSPNs salin: 0<br>iSPNs salin: 0<br>dSPNs amphet: 0<br>iSPNs amphet: 0.0228
Supp. Fig. 8d	SVM prediction error – effect of amphetamine	Salin<br>dSPNs: 10 sessions in 6 mice<br>iSPNs: 13 sessions in 9 mice<br>Amphet<br>dSPNs: 6 sessions in 6 mice<br>iSPNs: 9 sessions in 9 mice	Linear mixed effect model followed by ANOVA (Statterthwaite degrees of freedom)	DF1 DF2 F p-val<br>(Intercept) 1 17.08 3015.1 1.2e-20<br>genotype 1 17.08 3.385 0.0832<br>drug 1 17.195 12.224 0.0027<br>shuffle 1 17.885 118.56 2.6e-9<br>genotype:drug 1 17.195 2.4975 0.1323<br>genotype:shuffle 1 17.885 3.207 0.0902<br>drug:shuffle 1 26.549 43.871 4.5e-7<br>genotype:drug:shuffle 1 26.549 0.674 0.433	Permutation-based t-test between genotypes for and paired t-test between observed data and shuffle (corrected for multiple comparisons)	dSPNs vs. iSPNs<br>salin: 0.241<br>amphet: 0.0112<br><br>salin vs. amphet<br>dSPNs: 0.0128<br>iSPNs: 0<br><br>observed vs. shuffle<br>dSPNs salin: 0<br>iSPNs salin: 0<br>dSPNs amphet: 0<br>iSPNs amphet: 0.0128
Fig. 5b (left)	Distribution of significance of BI	dSPNs: 29 sessions in 8 mice<br>iSPNs: 37 sessions in 9 mice	Linear mixed effect model followed by ANOVA (Statterthwaite degrees of freedom)	DF1 DF2 F p-val<br>(Intercept) 1 18.07 1012.9 2.5e-17<br>genotype 1 18.07 4.282 0.053<br>bins 1 17.787 338.17 5.1e-13<br>genotype:bins 1 17.787 4.603 0.046		
Fig. 5b (right)	Average proportion behavior-active	dSPNs: 29 sessions in 8 mice<br>iSPNs: 37 sessions in 9 mice	Permutation-based t-test	dSPNs vs. iSPNs: p = 0		
Supp. Fig. 10d (left)	Distribution behavior-active per behavior	dSPNs: 29 sessions in 8 mice<br>iSPNs: 37 sessions in 9 mice	Linear mixed effect model followed by ANOVA (Statterthwaite degrees of freedom)	DF1 DF2 F p-val<br>(Intercept) 1 17.993 285.7 1.7e-12<br>genotype 1 17.993 8.4162 0.0095<br>behaviors 11 23.711 20.846 1.4e-9<br>genotype:behaviors 11 23.711 5.1714 0.0004		
Supp. Fig. 11a (right)	Distribution behavior-active per number of behaviors	dSPNs: 29 sessions in 8 mice<br>iSPNs: 37 sessions in 9 mice	Linear mixed effect model followed by ANOVA (Statterthwaite degrees of freedom)	DF1 DF2 F p-val<br>(Intercept) 1 18.487 309.51 6.9e-14<br>genotype 1 18.487 7.7670 0.0112<br>number 1 17.802 79.68 5.5e-8<br>genotype:number 1 17.802 8.4187 0.0096		
Fig. 5c<br>Supp. Fig. 11b	SVM prediction accuracy – behavior-active cells	dSPNs: 29 sessions in 8 mice<br>iSPNs: 37 sessions in 9 mice	Linear mixed effect model followed by ANOVA (Statterthwaite degrees of freedom)	DF1 DF2 F p-val<br>(Intercept) 1 17.63 537.97 1.2e-14<br>genotype 1 17.63 0.36061 0.556<br>tuning 1 16.86 6.3368 0.0222<br>shuffle 1 17.083 227.79 2.6e-11<br>genotype:tuning 1 16.86 8.6218 0.0093<br>genotype:shuffle 1 17.083 0.1737 0.682<br>tuning:shuffle 1 16.991 7.2919 0.0152<br>genotype:tunung:shuffle 1 16.991 7.1548 0.0160	Permutation-based t-test between genotypes and paired t-test between observed data and shuffle and between active vs. non-active (corrected for multiple comparisons)	dSPNs vs. iSPNs<br>active: 0.0018<br>non-active: 0.0728<br><br>active vs. non-active<br>dSPNs: 0<br>iSPNs: 0.3986<br><br>observed vs. shuffle<br>dSPNs active: 0<br>iSPNs active: 0<br>dSPNs non-active: 0<br>iSPNs non-active: 0
Fig. 5d<br>Supp. Fig. 11c	SVM prediction error – behavior-active cells	dSPNs: 29 sessions in 8 mice<br>iSPNs: 37 sessions in 9 mice	Linear mixed effect model followed by ANOVA (Statterthwaite degrees of freedom)	DF1 DF2 F p-val<br>(Intercept) 1 17.635 2566 1.5e-20<br>genotype 1 17.635 0.05887 0.811<br>tuning 1 14.715 8.415 0.0112<br>shuffle 1 14.77 277.09 5.6e-11<br>genotype:tuning 1 14.715 9.847 0.00690<br>genotype:shuffle 1 14.77 1.5629 0.231<br>tuning:shuffle 1 15.468 11.647 0.00371<br>genotype:tunung:shuffle 1 15.468 7.096 0.01735	Permutation-based t-test between genotypes and paired t-test between observed data and shuffle and between active vs. non-active (corrected for multiple comparisons)	dSPNs vs. iSPNs<br>active: 0.001<br>non-active: 0.0934<br><br>active vs. non-active<br>dSPNs: 0<br>iSPNs: 0.296<br><br>observed vs. shuffle<br>dSPNs active: 0<br>iSPNs active: 0<br>dSPNs non-active: 0<br>iSPNs non-active: 0
Supp. Fig. 11d	SVM prediction accuracy – behavior-active cells, effect of significance threshold	dSPNs: 29 sessions in 8 mice<br>iSPNs: 37 sessions in 9 mice	Linear mixed effect model followed by ANOVA (Statterthwaite degrees of freedom)	DF1 DF2 F p-val<br>(Intercept) 1 18.397 1710.7 1.3e-19<br>genotype 1 18.397 0.75634 0.39568<br>tuning 1 17.718 11.358 0.00347<br>threshold 4 333.44 0.92857 0.44743<br>genotype:tuning 1 17.718 7.8384 0.01197<br>genotype:threshold 4 333.44 0.35859 0.83799<br>tuning:threshold 4 234.02 15.593 2.5e-11<br>genotype:tunung:threshold 4 234.02 0.34849 0.84493	Permutation-based t-test between genotypes and between active vs. non-active (corrected for multiple comparisons)	dSPNs vs. iSPNs<br>active | non-active<br>BI=3: 0.0002 | 0.214<br>BI=3.5: 0.0012 | 0.0652<br>BI=4: 0.0026 | 0.0584<br>BI=4.5: 0.0074 | 0.231<br>BI=5: 0.0148 | 0.0338<br><br>active vs. non-active<br>dSPNs | iSPNs<br>BI=3: 0 | 0.026<br>BI=3.5:0 | 0.1816

						BI=4: 0.0002 | 0.3962<br>BI=4.5: 0.002 | 0.2046<br>BI=5: 0.0011 | 0.0434
Supp. Fig. 12b (left)	Average proportion behavior-excited	dSPNs:<br>29 sessions in 8 mice<br>iSPNs:<br>37 sessions in 9 mice	Permutation-based t-test	dSPNs vs. iSPNs: p = 0		
Supp. Fig. 12b (right)	Distribution behavior-excited per behavior	dSPNs:<br>29 sessions in 8 mice<br>iSPNs:<br>37 sessions in 9 mice	Linear mixed effect model followed by ANOVA (Statterthwaite degrees of freedom)	DF1 DF2 F p-val<br>(Intercept) 1 20.011 6.2201 0.0215<br>genotype 1 20.011 0.0053 0.9422<br>behaviors 11 26.308 14.444 1.2e-8<br>genotype:behaviors 11 26.308 3.2242 0.0067		
Supp. Fig. 12c	Comparison neurons classified as behavior-active and classified as behavior-excited (Jaccard index)	dSPNs:<br>29 sessions in 8 mice<br>iSPNs:<br>37 sessions in 9 mice	Linear mixed effect model followed by ANOVA (Statterthwaite degrees of freedom)	DF1 DF2 F p-val<br>(Intercept) 1 17.767 1258.4 6.1e-18<br>genotype 1 17.767 9.2089 0.0072<br>shuffle 1 19.154 5402.1 6.0e-25<br>genotype:shuffle 1 19.154 1.7361 0.2032	Permutation-based t-test between conditions (corrected for multiple comparisons)	observed vs. shuffle<br>dSPNs: 0<br>iSPNs: 0
Supp. Fig. 12d	SVM prediction accuracy – behavior-excited cells	dSPNs:<br>29 sessions in 8 mice<br>iSPNs:<br>37 sessions in 9 mice	Linear mixed effect model followed by ANOVA (Statterthwaite degrees of freedom)	DF1 DF2 F p-val<br>(Intercept) 1 12.268 394.03 1.1e-10<br>genotype 1 12.268 2.7891 0.120<br>tuning 1 11.08 28.956 0.0002<br>genotype:tuning 1 11.08 18.327 0.00128	Permutation-based t-test between genotypes and paired t-test between observed data and shuffle and between active vs. non-active (corrected for multiple comparisons)	dSPNs vs. iSPNs<br>excited: 0.0002<br>non-excited: 0.0354<br><br>active vs. non-active<br>dSPNs: 0<br>iSPNs: 0.0966
Supp. Fig. 16c	SVM prediction number of cells – behavior active cells	dSPNs:<br>29 sessions in 8 mice<br>iSPNs:<br>37 sessions in 9 mice	Linear mixed effect model followed by ANOVA (Statterthwaite degrees of freedom)	DF1 DF2 F p-val<br>(Intercept) 1 17.77 93.64 1.6e-8<br>genotype 1 17.77 0.1392 0.714<br>tuning 1 16.246 0.7391 0.402<br>genotype:tuning 1 16.246 3.1595 0.0942	Permutation-based t-test between conditions (corrected for multiple comparisons)	dSPNs vs. iSPNs<br>active: 0.125<br>non-active: 0.0204<br><br>active vs. non-active<br>dSPNs: 0.232<br>iSPNs: 0.003
Supp. Fig. 16d	SVM prediction accuracy – same number of cells	dSPNs:<br>29 sessions in 8 mice<br>iSPNs:<br>37 sessions in 9 mice	Linear mixed effect model followed by ANOVA (Statterthwaite degrees of freedom)	DF1 DF2 F p-val<br>(Intercept) 1 18.01 579.4 3.8e-15<br>genotype 1 18.01 1.663 0.213<br>tuning 1 73.59 14.21 0.0003<br>shuffle 1 16.03 202.4 1.7e-10<br>genotype:tuning 1 73.59 39.53 2.1e-8<br>genotype:shuffle 1 16.03 3.165 0.0942<br>tuning:shuffle 1 49.84 52.55 2.5e-9<br>genotype:tunung:shuffle 1 49.84 5.098 0.0284	Permutation-based t-test between genotypes and paired t-test between observed data and shuffle and between active vs. non-active (corrected for multiple comparisons)	dSPNs vs. iSPNs<br>active: 0.0002<br>non-active: 0.4746<br><br>active vs. non-active<br>dSPNs: 0.0004<br>iSPNs: 0.2656<br><br>observed vs. shuffle<br>dSPNs active: 0<br>iSPNs active: 0<br>dSPNs non-active: 0<br>iSPNs non-active: 0
Supp. Fig. 16e	SVM prediction error – same number of cells	dSPNs:<br>29 sessions in 8 mice<br>iSPNs:<br>37 sessions in 9 mice	Linear mixed effect model followed by ANOVA (Statterthwaite degrees of freedom)	DF1 DF2 F p-val<br>(Intercept) 1 17.93 2547 8.8e-21<br>genotype 1 17.93 0.9773 0.336<br>tuning 1 380.0 5.849 0.161<br>shuffle 1 107.5 193.6 8.7e-26<br>genotype:tuning 1 380.0 12.92 0.0004<br>genotype:shuffle 1 107.5 4.780 0.0310<br>tuning:shuffle 1 106.3 21.94 8.4e-6<br>genotype:tunung:shuffle 1 106.3 1.971 0.163	Permutation-based t-test between genotypes and paired t-test between observed data and shuffle and between active vs. non-active (corrected for multiple comparisons)	dSPNs vs. iSPNs<br>active: 0.0004<br>non-active: 0.404<br><br>active vs. non-active<br>dSPNs: 0<br>iSPNs: 0.2812<br><br>observed vs. shuffle<br>dSPNs active: 0<br>iSPNs active: 0<br>dSPNs non-active: 0<br>iSPNs non-active: 0
Supp. Fig. 16f	SVM prediction number of cells – same number of cells	dSPNs:<br>29 sessions in 8 mice<br>iSPNs:<br>37 sessions in 9 mice	Linear mixed effect model followed by ANOVA (Statterthwaite degrees of freedom)	DF1 DF2 F p-val<br>(Intercept) 1 16.42 79.42 1.1e-7<br>genotype 1 16.42 0.0615 0.807<br>tuning 1 82.72 3.0e-15 1<br>genotype:tuning 1 82.72 2.1e-17 1	Permutation-based t-test between conditions (corrected for multiple comparisons)	dSPNs vs. iSPNs<br>active: 0.387<br>non-active: 0.384<br><br>active vs. non-active<br>dSPNs: 0.489<br>iSPNs: 0.495
Fig. 6a (right)	Distribution of activation occurrence	dSPNs:<br>29 sessions in 8 mice<br>iSPNs:<br>37 sessions in 9 mice	Linear mixed effect model followed by ANOVA (Statterthwaite degrees of freedom)	DF1 DF2 F p-val<br>(Intercept) 1 7300 10677 0<br>genotype 1 7300 8.4682 0.00362<br>bins 1 7300 6887.2 0<br>genotype:bins 1 7300 11.348 0.00076		
Fig. 6b (left)	Distribution behavior-silent per behavior	dSPNs:<br>29 sessions in 8 mice<br>iSPNs:<br>37 sessions in 9 mice	Linear mixed effect model followed by ANOVA (Statterthwaite degrees of freedom)	DF1 DF2 F p-val<br>(Intercept) 1 18.005 56.527 5.9e-7<br>genotype 1 18.005 10.035 0.0053<br>behaviors 10 30.344 7.905 4.3e-6<br>genotype:behaviors 10 30.344 1.285 0.283		
Fig. 6b (right)	Distribution behavior-silent per number of behaviors	dSPNs:<br>29 sessions in 8 mice<br>iSPNs:<br>37 sessions in 9 mice	Linear mixed effect model followed by ANOVA (Statterthwaite degrees of freedom)	DF1 DF2 F p-val<br>(Intercept) 1 434 62.153 2.6e-14<br>genotype 1 434 0.022 0.884<br>number 1 434 0.024 0.878<br>genotype:number 1 434 0.159 0.691		
Fig. 6c	SVM prediction – behavior-silent cells	dSPNs:<br>29 sessions in 8 mice<br>iSPNs:<br>37 sessions in 9 mice	Linear mixed effect model followed by ANOVA (Statterthwaite degrees of freedom)	DF1 DF2 F p-val<br>(Intercept) 1 12.461 22310 1.2e-21<br>genotype 1 12.461 0.3953 0.541<br>tuning 1 16.765 0.0270 0.871<br>behavior 11 27.705 16.993 1.6e-9<br>genotype:tuning 1 16.765 0.7274 0.411<br>genotype:behavior 11 27.705 1.676 0.011<br>tuning:behavior 11 38.522 2.7010 0.132	Permutation-based paired t-test between silent vs. non-silent for each genotype (corrected for multiple comparisons)	silent vs. non-silent:<br>dSPNs | iSPNs<br>l. fast: 0.014 | 0.0018<br>l. str: 0.236 | 0.0072<br>l. right: 0.452 | 0.0326<br>l. left: 0.346 | 0.0394<br>st. right: 0.472 | 0<br>st. left: 0.067 | 0

				genotype:tuning:behavior 11 38.522 0.591 0.842		head up: 0.102 | 0.458<br>rear.: 0.36 | 0.317<br>groom.: 0.3 | 0.0826<br>st. sniff.: 0.126 | 0.303<br>l. sniff.: 0.425 | 0<br>immo.: 0 | 0.0176
Supp. Fig. 16g	SVM number of cells used – behavior-silent cells	dSPNs:<br>29 sessions in 8 mice<br>iSPNs:<br>37 sessions in 9 mice	Linear mixed effect model followed by ANOVA (Statterthwaite degrees of freedom	DF1 DF2 F p-val<br>(Intercept) 1 18.239 90.71 1.7e-8<br>genotype 1 18.239 0.794 0.412<br>tuning 1 18.349 0.588 3.9e-7<br>behavior 11 56.538 0.954 0.498<br>genotype:tuning 1 18.349 0.192 0.667<br>genotype:behavior 11 56.538 0.533 0.873<br>tuning:behavior 11 36.648 3.860 0.0009<br>genotype:tuning:behavior 11 36.648 2.240 0.335	Permutation-based paired t-test between silent vs. non-silent for each genotype (corrected for multiple comparisons)	silent vs. non-silent:<br>for all behaviors<br>dSPNs: p = 0<br>iSPNs: p = 0<br><br>dSPNs vs. iSPNs<br>silent | non-silent<br>l. fast: 0 | 0.197<br>l. str: 0 | 0.0026<br>l. right: 0 | 0.0788<br>l. left: 0 | 0.0292<br>st. right: 0 | 0.964<br>st. left: 0 | 0.047<br>head up: 0 | 0.248<br>rear.: 0 | 0.225<br>groom.: 0 | 0.235<br>st. sniff.: 0 | 0.147<br>l. sniff.: 0 | 0.0048<br>immo.: 0 | 0.063
Supp. Fig. 16h	SVM number of cells used – behavior silent cells, same number of cells	dSPNs:<br>29 sessions in 8 mice<br>iSPNs:<br>37 sessions in 9 mice	Linear mixed effect model followed by ANOVA (Statterthwaite degrees of freedom	DF1 DF2 F p-val<br>(Intercept) 1 628 1216 7e-149<br>genotype 1 628 13.23 0.0003<br>tuning 1 628 5.0e-16 1<br>behavior 11 628 6.578 1.9e-10<br>genotype:tuning 1 628 2.0e-15 1<br>genotype:behavior 11 628 3.02 0.0006<br>tuning:behavior 11 628 2.8e-17 1<br>genotype:tuning:behavior 11 628 2.5e-14 1	Permutation-based paired t-test between silent vs. non-silent for each genotype (corrected for multiple comparisons)	silent vs. non-silent:<br>for all behaviors<br>dSPNs: p > 0.05<br>iSPNs: p > 0.05<br><br>dSPNs vs. iSPNs:<br>for all behaviors<br>silent: p > 0.05<br>non-silent: p > 0.05
Supp. Fig. 16i	SVM prediction – behavior-silent cells, same number of cells	dSPNs:<br>29 sessions in 8 mice<br>iSPNs:<br>37 sessions in 9 mice	Linear mixed effect model followed by ANOVA (Statterthwaite degrees of freedom	DF1 DF2 F p-val<br>(Intercept) 1 20.673 28571 1.2e-21<br>genotype 1 20.673 0.613 0.541<br>tuning 1 23.148 0.432 0.871<br>behavior 11 29.101 29.191 1.6e-9<br>genotype:tuning 1 23.147 0.461 0.411<br>genotype:behavior 11 29.101 2.138 0.132<br>tuning:behavior 11 28.944 1.884 0.0111<br>genotype:tuning:behavior 11 28.944 0.129 0.824	Permutation-based paired t-test between silent vs. non-silent for each genotype (corrected for multiple comparisons)	silent vs. non-silent:<br>dSPNs | iSPNs<br>l. fast: 0.0012 | 0.0024<br>l. str: 0.296 | 0.0332<br>l. right: 0.462 | 0.0215<br>l. left: 0.480 | 0.0408<br>st. right: 0.469 | 0<br>st. left: 0.073 | 0<br>head up: 0.064 | 0.258<br>rear.: 0.317 | 0.165<br>groom.: 0.298 | 0.295<br>st. sniff.: 0.179 | 0.383<br>l. sniff.: 0.416 | 0<br>immo.: 0.002 | 0.083
Supp. Fig. 14c (left)	Distribution shuffle behavior-silent per behavior	dSPNs:<br>29 sessions in 8 mice<br>iSPNs:<br>37 sessions in 9 mice	Linear mixed effect model followed by ANOVA (Statterthwaite degrees of freedom	DF1 DF2 F p-val<br>(Intercept) 1 18.138 85.27 2.8e-8<br>genotype 1 18.138 7.982 0.0112<br>behaviors 10 36.001 7.691 2.0e-6<br>genotype:behaviors 10 36.001 0.932 0.517		
Supp. Fig. 14c (right)	Distribution shuffle behavior-silent per number of behaviors	dSPNs:<br>29 sessions in 8 mice<br>iSPNs:<br>37 sessions in 9 mice	Linear mixed effect model followed by ANOVA (Statterthwaite degrees of freedom	DF1 DF2 F p-val<br>(Intercept) 1 438 54.93 6.5e-13<br>genotype 1 438 0.0237 0.875<br>number 1 438 0.0021 0.964<br>genotype:number 1 438 0.0418 0.838		
Supp. Fig. 14d	SVM prediction – shuffle behavior-silent cells	dSPNs:<br>29 sessions in 8 mice<br>iSPNs:<br>37 sessions in 9 mice	Linear mixed effect model followed by ANOVA (Statterthwaite degrees of freedom<br><br>separate analyses for dSPNs sessions and iSPNs sessions	dSPNs (restricted to behaviors: loco. fast, head up, groom, st. sniff., and immo.) DF1 DF2 F p-val<br>(Intercept) 1 4.46 3797.8 1.0e-7<br>tuning 1 6.16 0.3608 0.579<br>behavior 4 6.62 11.13 0.0045<br>tuning:behavior 4 6.09 4.846 0.4248<br><br>iSPNs (with all behaviors)<br>DF1 DF2 F p-val<br>(Intercept) 1 12.35 15640 2e-292<br>tuning 1 11.81 0.4670 0.495<br>behavior 11 14.38 17.325 3.0e-27<br>tuning:behavior 11 16.49 2.347 0.0084	Permutation-based paired t-test between silent vs. non-silent for each genotype (corrected for multiple comparisons)	silent vs. non-silent:<br>dSPNs | iSPNs<br>l. fast: 0.0044 | 0<br>l. str: *No data* | 0.0008<br>l. right: *No data* | 0.0284<br>l. left: 0.0401 | 0.013<br>st. right: *No data* | 0<br>st. left: *No data* | 0<br>head up: 0.117 | 0.218<br>rear.: *No data* | 0.237<br>groom.: 0.492 | 0.0648<br>st. sniff.: 0.161 | 0.285<br>l. sniff.: *No data* | 0<br>immo.: 0.0004 | 0.0086
Supp. Fig. 15b (left)	Distribution behavior-inactive per behavior	dSPNs:<br>29 sessions in 8 mice<br>iSPNs:<br>37 sessions in 9 mice	Linear mixed effect model followed by ANOVA (Statterthwaite degrees of freedom)	DF1 DF2 F p-val<br>(Intercept) 1 17.987 73.336 2.2e-8<br>genotype 1 17.987 7.364 0.0142<br>behaviors 10 29.659 12.536 3.8e-8<br>genotype:behaviors 10 29.659 1.831 0.098		
Supp. Fig. 15b (right)	Distribution behavior-inactive per behavior	dSPNs:<br>29 sessions in 8 mice<br>iSPNs:<br>37 sessions in 9 mice	Linear mixed effect model followed by ANOVA (Statterthwaite degrees of freedom)	DF1 DF2 F p-val<br>(Intercept) 1 438 76.464 4.8e-17<br>genotype 1 438 0.0055 0.941<br>number 1 438 0.0197 0.888<br>genotype:number 1 438 0.0589 0.808		
Supp. Fig. 15c	SVM prediction – behavior-inactive cells	dSPNs:<br>29 sessions in 8 mice<br>iSPNs:<br>37 sessions in 9 mice	Linear mixed effect model followed by ANOVA (Statterthwaite degrees of freedom)	DF1 DF2 F p-val<br>(Intercept) 1 12.555 24879 4.4e-22<br>genotype 1 12.555 0.0042 0.949<br>tuning 1 33.514 5.0017 0.032<br>behavior 11 25.632 25.982 3.8e-11<br>genotype:tuning 1 33.514 0.5182 0.477<br>genotype:behavior 11 25.635 0.6416 0.777<br>tuning:behavior 11 23.951 0.7157 0.713<br>genotype:tuning:behavior 11 23.951 0.3642 0.958	Permutation-based paired t-test between inactive vs. non-inactive for each genotype (corrected for multiple comparisons)	inactive vs. non-inactive:<br>dSPNs | iSPNs<br>l. fast: 0.0008 | 0.0004<br>l. str: 0.214 | 0.0006<br>l. right: 0.337 | 0.0054<br>l. left: 0.335 | 0.0401<br>st. right: 0.158 | 0<br>st. left: 0.005 | 0<br>head up: 0.238 | 0.487<br>rear.: 0.320 | 0.136<br>groom.: 0.410 | 0.329<br>st. sniff.: 0.131 | 0.327<br>l. sniff.: 0.256 | 0<br>immo.: 0.354 | 0.449

Supp. Fig. 17b (left)	Distribution of registration score	dSPNs:<br>8 mice<br>iSPNs:<br>9 mice	Linear mixed effect model followed by ANOVA (Statterthwaite degrees of freedom)	DF1 DF2 F p-val<br>(Intercept) 1 209 46.304 1.0e-10<br>genotype 1 209 0.00448 0.947<br>bins_score 1 209 198.16 4.3e-32<br>genotype:bins_score 1 209 0.00212 0.963		
Supp. Fig. 17b (right)	Average registration score	dSPNs:<br>8 mice<br>iSPNs:<br>9 mice	Permutation-based t-test	dSPNs vs. iSPNs: p = 0.645		
Supp. Fig. 17c	Number of registered neurons	dSPNs:<br>72 pairs of sessions in 8 mice<br>iSPNs:<br>80 pairs of sessions in 9 mice	Linear mixed effect model followed by ANOVA (Statterthwaite degrees of freedom)	DF1 DF2 F p-val<br>(Intercept) 1 18.057 68.497 1.5e-7<br>genotype 1 18.057 5.5e-6 0.998<br>days 1 135.89 47.906 1.6e-10<br>genotype:days 1 135.89 1.196 0.276		
Fig. 7a Supp. Fig. 17d-e	Neuronal activation similarity during behaviors across days and shuffle controls	dSPNs:<br>52 pairs of sessions in 8 mice<br>iSPNs:<br>62 pairs of sessions in 9 mice	Linear mixed effect model followed by ANOVA (Statterthwaite degrees of freedom)	DF1 DF2 F p-val<br>(Intercept) 1 17.948 516.12 1.1e-14<br>genotype 1 17.948 8.2278 0.0102<br>behavior 11 3702.9 47.02 2.8e-97<br>shuffle 2 18.28 93.26 2.6e-10<br>days 1 14.623 18.173 0.00072<br>genotype:behavior 11 3702.9 31.909 2.5e-65<br>genotype:shuffle 2 18.28 2.4415 0.1149<br>behavior:shuffle 22 3705.6 1.7385 0.0176<br>genotype:days 1 14.623 0.0295 0.866<br>behavior:days 11 3702.7 3.0913 0.00038<br>shuffle:days 2 27.038 14.498 5.3e-5<br>genotype:behavior:shuffle 22 3705.6 0.6570 0.884<br>genotype:behavior:days 11 3702.7 13.067 9.4e-25<br>genotype:shuffle:days 2 27.038 0.8895 0.423<br>behavior:shuffle:days 22 3703.3 0.5137 0.970<br>geno:behav:shuffle:days 22 3703.3 0.6286 0.907	Analysis of covariance for each behavior between genotypes and for each genotype between observed data and respective shuffle across days<br><br>[only main effect between genotype or between real and shuffled data is reported]	dSPNs vs. iSPNs<br>l. fast: $F_{1,95}$=74.4; p=0<br>l. str: $F_{1,116}$=85.1; p=0<br>l. right: $F_{1,114}$=87.5;p=0<br>l. left: $F_{1,113}$=60.4;p=0<br>st. right: $F_{1,111}$=120.1; p=0<br>st. left: $F_{1,113}$=67.7;p=0<br>head up:$F_{1,114}$=54.2;p=0<br>rear.: $F_{1,90}$=32.1;p=0<br>groom.: $F_{1,110}$=9.51; p=0.0026<br>st. sniff.: $F_{1,116}$=28.1;p=0<br>l. sniff.: $F_{1,114}$=73.2;p=0<br>immo.: $F_{1,107}$=2.87; p=0.0929<br><br>dSPNs, real vs. shuffle<br>l. fast: $F_{1,81}$=220.3;p=0<br>l. str: $F_{1,94}$=236.7;p=0<br>l. right: $F_{1,94}$=148.0;p=0<br>l. left: $F_{1,92}$=167.9;p=0<br>st. right: $F_{1,94}$=193.0;p=0<br>st. left: $F_{1,94}$=135.8;p=0<br>head up: $F_{1,94}$=213.3; p=0<br>rear.: $F_{1,88}$=280.0;p=0<br>groom.: $F_{1,94}$=174.4;p=0<br>st. sniff.: $F_{1,94}$=158.7; p=0<br>l. sniff.: $F_{1,92}$=49.7;p=0<br>immo.: $F_{1,88}$=47.2;p=0<br><br>iSPNs, real vs. shuffle<br>l. fast: $F_{1,100}$=31.7;p=0<br>l. str: $F_{1,125}$=47.6;p=0<br>l. right: $F_{1,121}$=24.5;p=0<br>l. left: $F_{1,121}$=30.2;p=0<br>st. right: $F_{1,115}$=39.0;p=0<br>st. left: $F_{1,119}$=18.0;p=0<br>head up:$F_{1,121}$=59.4;p=0<br>rear.: $F_{1,80}$=28.5;p=0<br>groom.: $F_{1,113}$=50.0;p=0<br>st. sniff.: $F_{1,125}$=34.9;p=0<br>l. sniff.: $F_{1,124}$=11.4; p=0.001<br>immo.: $F_{1,113}$=32.0;p=0<br><br>dSPNs, real vs. neighbor<br>l. fast: $F_{1,81}$=66.1;p=0<br>l. str: $F_{1,94}$=81.3;p=0<br>l. right: $F_{1,94}$=60.07;p=0<br>l. left: $F_{1,92}$=119.87;p=0<br>st. right: $F_{1,94}$=100.5;p=0<br>st. left: $F_{1,94}$=74.7;p=0<br>head up: $F_{1,94}$=101.3; p=0<br>rear.: $F_{1,88}$=151.8;p=0<br>groom.: $F_{1,94}$=51.7;p=0<br>st. sniff.: $F_{1,94}$=75.9;p=0<br>l. sniff.: $F_{1,92}$=30.2;p=0<br>immo.: $F_{1,88}$=18.9;p=0<br><br>iSPNs, real vs. neighbor<br>l. fast: $F_{1,100}$=10.4; p=0.0017<br>l. str: $F_{1,125}$=20.3;p=0<br>l. right: $F_{1,121}$=6.99; p=0.0093<br>l. left: $F_{1,121}$=12.92; p=0.0005<br>st. right: $F_{1,115}$=6.52; p=0.012<br>st. left: $F_{1,119}$=9.93; p=0.0021<br>head up: $F_{1,121}$=27.0; p=0<br>rear.: $F_{1,80}$=9.56; p=0.0027<br>groom.: $F_{1,113}$=18.2;p=0<br>st. sniff.: $F_{1,125}$=13.5; p=0.0004<br>l. sniff.: $F_{1,124}$=4.12; p=0.0446<br>immo.: $F_{1,113}$=11.6; p=0.0009

Fig. 7b (left)	Stability coding behavior-active	dSPNs:<br>58 pairs of sessions<br>iSPNs:<br>70 pairs of sessions	Linear mixed effect model followed by ANOVA (Statterthwaite degrees of freedom)	DF1 DF2 F p-val<br>(Intercept) 1 12.157 150.5 1.3e-8<br>genotype 1 12.157 9.8404 0.00846<br>days 1 14.572 10.226 0.00618<br>genotype:days 1 14.572 0.0022 0.963	Permutation-based t-test between genotypes	dSPNs vs. iSPNs: 0
Fig. 7b (right)	Stability coding behavior-silent	dSPNs:<br>16 pairs of sessions<br>iSPNs:<br>34 pairs of sessions	Linear mixed effect model followed by ANOVA (Statterthwaite degrees of freedom)	DF1 DF2 F p-val<br>(Intercept) 1 94 209.45 1.2e-25<br>genotype 1 94 0.6534 0.421<br>days 1 94 0.1673 0.685<br>genotype:days 1 94 5.389 0.0224	Permutation-based t-test between genotypes	dSPNs vs. iSPNs: 0.014
Supp. Fig. 18c	Stability coding behavior-inactive	dSPNs:<br>16 pairs of sessions<br>iSPNs:<br>29 pairs of sessions	Linear mixed effect model followed by ANOVA (Statterthwaite degrees of freedom)	DF1 DF2 F p-val<br>(Intercept) 1 88 406.8 9.4e-35<br>genotype 1 88 0.2615 0.610<br>days 1 88 0.0422 0.838<br>genotype:days 1 88 7.936 0.00598	Permutation-based t-test between genotypes	dSPNs vs. iSPNs: 0.006
Fig. 7c<br>Supp. Fig. 18a	Longitudinal SVM prediction – accuracy	dSPNs:<br>121 pairs of sessions<br>iSPNs:<br>171 pairs of sessions	Linear mixed effect model followed by ANOVA (Statterthwaite degrees of freedom)	DF1 DF2 F p-val<br>(Intercept) 1 17.687 1466.7 1.8e-18<br>genotype 1 17.867 14.891 0.00118<br>days 1 12.053 27.347 0.00021<br>shuffle 1 17.228 155.01 4.8e-10<br>genotype:days 1 12.053 0.0312 0.8627<br>genotype:shuffle 1 17.228 3.309 0.0863<br>days:shuffle 1 26.479 46.764 6.7e-7<br>genotype:days:shuffle 1 26.479 0.2331 0.6332	Analysis of covariance between genotypes and for each genotype between observed data and respective shuffle across days	dSPNs vs. iSPNs:<br>Genotype<br>$F_{1,269}$=59.85; p=2.1e-13<br>Days<br>$F_{1,269}$=20.73; p=8.0e-6<br>Genotype:days<br>$F_{1,269}$=0.45; p=0.5046<br><br>dSPNs vs. shuffle<br>Shuffle<br>$F_{1,238}$=657.58; p=0<br>Days<br>$F_{1,238}$=18.12; p=3.0e-5<br>Shuffle:days<br>$F_{1,238}$=17.58; p=3.9e-5<br><br>iSPNs vs. shuffle<br>Shuffle<br>$F_{1,300}$=313.85; p=0<br>Days<br>$F_{1,300}$=2.59; p=0.1084<br>Shuffle:days<br>$F_{1,300}$=3.17; p=0.0762
Supp. Fig. 18b	Longitudinal SVM prediction – error	dSPNs:<br>121 pairs of sessions<br>iSPNs:<br>171 pairs of sessions	Linear mixed effect model followed by ANOVA (Statterthwaite degrees of freedom)	DF1 DF2 F p-val<br>(Intercept) 1 17.485 4992.3 2.4e-23<br>genotype 1 17.485 7.5379 0.0134<br>days 1 12.933 0.8135 0.780<br>shuffle 1 17.453 185.39 2.0e-10<br>genotype:days 1 12.933 0.0797 0.7822<br>genotype:shuffle 1 17.453 8.524 0.0097<br>days:shuffle 1 21.686 15.047 3.6e-4<br>genotype:days:shuffle 1 21.686 0.6216 0.4348	Analysis of covariance between genotypes and for each genotype between observed data and respective shuffle across days	dSPNs vs. iSPNs:<br>Genotype<br>$F_{1,257}$=67.7; p=9.5e-15<br>Days<br>$F_{1,257}$=0.15; p=0.6965<br>Genotype:days<br>$F_{1,257}$=1.31; p=0.2541<br><br>dSPNs vs. shuffle<br>Shuffle<br>$F_{1,238}$=452.09; p=0<br>Days<br>$F_{1,238}$=0.37; p=0.544<br>Shuffle:days<br>$F_{1,238}$=5.15; p=0.024<br><br>iSPNs vs. shuffle<br>Shuffle<br>$F_{1,300}$=161.22; p=0<br>Days<br>$F_{1,300}$=2.57; p=0.1098<br>Shuffle:days<br>$F_{1,300}$=0.82; p=0.3645
Fig. 8a	Longitudinal SVM prediction – behavior-active neurons	dSPNs:<br>93 pairs of sessions<br>iSPNs:<br>132 pairs of sessions	Linear mixed effect model followed by ANOVA (Statterthwaite degrees of freedom)	DF1 DF2 F p-val<br>(Intercept) 1 17.181 256.02 9.3e-12<br>genotype 1 17.181 0.42601 0.5226<br>days 1 22.194 13.439 0.00134<br>tuning 1 420.37 14.018 0.00020<br>genotype:days 1 22.194 0.22554 0.5984<br>genotype:tuning 1 420.37 22.161 3.4e-6<br>days:tuning 1 115.6 0.00314 0.9555<br>genotype:days:tuning 1 115.6 2.4326 0.1216	Analysis of covariance between active vs. non-active for each genotype across days	Active vs. non active<br><br>dSPNs<br>Tuning<br>$F_{1,208}$=56.24; p=1.8e-12<br>Days<br>$F_{1,208}$=14.87; p=0.00015<br>Tuning:days<br>$F_{1,208}$=3.62; p=0.0633<br><br>iSPNs<br>Tuning<br>$F_{1,250}$=0.02; p=0.8757<br>Days<br>$F_{1,250}$=4.11; p=0.0438<br>Tuning:days<br>$F_{1,250}$=0.92; p=0.228
Fig. 8b	Longitudinal SVM prediction – behavior-silent neurons	Ambulatory behaviors<br>dSPNs:<br>18 pairs of sessions<br>iSPNs:<br>52 pairs of sessions<br><br>Static behaviors<br>dSPNs:<br>46 pairs of sessions<br>iSPNs:<br>69 pairs of sessions	Linear mixed effect model followed by ANOVA (Statterthwaite degrees of freedom)	DF1 DF2 F p-val<br>(Intercept) 1 17.934 1811.1 1.8e-19<br>genotype 1 17.934 0.5246 0.478<br>days 1 338.5 0.7528 0.386<br>tuning 1 93.535 0.2306 0.6322<br>behavior_class 1 18.202 34.251 1.5e-5<br>genotype:days 1 338.5 0.2102 0.647<br>genotype:tuning 1 93.535 0.6614 0.418<br>genotype:behav_class 1 18.202 0.00016 0.990<br>days:tuning 1 595.99 0.1637 0.686<br>days:behav_class 1 171.43 0.00235 0.961<br>tuning:behav_class 1 37.418 8.0138 0.0074<br>geno:days:tuning 1 595.99 0.0552 0.814<br>geno:days:behav_class 1 171.43 0.00440 0.947<br>geno:tuning:behav_class 1 37.418 1.7866 0.189<br>days:tuning:behav_class 1 419.49 0.19102 0.662<br>geno:days:tuning:behav_class 1 419.49 0.19477 0.659	Analysis of covariance between silent vs. non-silent for each genotype for each behavior_class (ie ambulatory or static behaviors)	Silent vs. non-silent<br><br>Static behaviors<br>dSPNs<br>Tuning<br>$F_{1,68}$=0.03; p=0.243<br>Days<br>$F_{1,68}$=20.51; p=0<br>Tuning:days<br>$F_{1,68}$=0.02; p=0.902<br>iSPNs<br>Tuning<br>$F_{1,310}$=1.03; p=0.311<br>Days<br>$F_{1,310}$=0.242; p=0.623<br>Tuning:days<br>$F_{1,310}$=0.765; p=0.382<br><br>Ambulatory behaviors<br>dSPNs<br>Tuning

						$F_{1,34}$=2.07; p=0.160<br>Days<br>$F_{1,34}$=0.01; p=0.907<br>Tuning:days<br>$F_{1,34}$=0.01; p=0.906<br>iSPNs<br>Tuning<br>$F_{1,318}$=344.0; p=1.4e-52<br>Days<br>$F_{1,318}$=4.43; p=0.036<br>Tuning:days<br>$F_{1,318}$=0.795; p=0.373
Supp. Fig. 18d	Longitudinal SVM prediction – behavior-inactive neurons	Ambulatory behaviors<br>dSPNs:<br>20 pairs of sessions<br>iSPNs:<br>46 pairs of sessions<br><br>Static behaviors<br>dSPNs:<br>67 pairs of sessions<br>iSPNs:<br>75 pairs of sessions	Linear mixed effect model followed by ANOVA (Statterthwaite degrees of freedom)	DF1 DF2 F p-val<br>(Intercept) 1 40.899 2488.9 2.9e-38<br>genotype 1 40.899 3.358 0.0742<br>days 1 13.683 3.150 0.0982<br>tuning 1 111.21 9.411 0.0027<br>behavior_class 1 63.662 54.393 4.2e-10<br>genotype:days 1 13.683 0.00223 0.963<br>genotype:tuning 1 111.21 0.1293 0.720<br>genotype:behav_class 1 63.662 0.6872 0.410<br>days:tuning 1 58.308 0.01869 0.892<br>days:behav_class 1 10.660 2.265 0.161<br>tuning:behav_class 1 43.494 1.215 0.276<br>geno:days:tuning 1 58.308 0.02839 0.867<br>geno:days:behav_class 1 10.660 1.1067 0.316<br>geno:tuning:behav_class 1 43.494 0.16206 0.689<br>days:tuning:behav_class 1 73.776 0.37797 0.541<br>geno:days:tuning:behav_class 1 73.776 0.57587 0.450	Analysis of covariance between inactive vs. non-inactive for each genotype for each behavior_class (ie ambulatory or static behaviors)	Inactive vs. non-inactive<br><br>Static behaviors<br>dSPNs<br>Tuning<br>$F_{1,132}$=2.79; p=0.0973<br>Days<br>$F_{1,132}$=5.14; p=0.025<br>Tuning:days<br>$F_{1,132}$=0.04; p=0.845<br>iSPNs<br>Tuning<br>$F_{1,404}$=3.53; p=0.0611<br>Days<br>$F_{1,404}$=1.42; p=0.233<br>Tuning:days<br>$F_{1,404}$=0.61; p=0.434<br><br>Ambulatory behaviors<br>dSPNs<br>Tuning<br>$F_{1,90}$=2.79; p=0.098<br>Days<br>$F_{1,90}$=0; p=0.961<br>Tuning:days<br>$F_{1,90}$=0.26; p=0.6131<br>iSPNs<br>Tuning<br>$F_{1,192}$=60.73; p=4.0e-13<br>Days<br>$F_{1,192}$=2.74; p=0.0994<br>Tuning:days<br>$F_{1,192}$=0.03; p=0.866

Supplementary references:

1. Mink, J.W. The basal ganglia: focused selection and inhibition of competing motor programs. *Prog Neurobiol* **50**, 381-425 (1996).
2. Parker, J.G., *et al.* Diametric neural ensemble dynamics in parkinsonian and dyskinetic states. *Nature* **557**, 177-182 (2018).
3. Durieux, P.F., Schiffmann, S.N. & de Kerchove d'Exaerde, A. Differential regulation of motor control and response to dopaminergic drugs by D1R and D2R neurons in distinct dorsal striatum subregions. *EMBO J* **31**, 640-653 (2012).
4. Kravitz, A.V., *et al.* Regulation of parkinsonian motor behaviours by optogenetic control of basal ganglia circuitry. *Nature* **466**, 622-626 (2010).
5. Yttri, E.A. & Dudman, J.T. Opponent and bidirectional control of movement velocity in the basal ganglia. *Nature* **533**, 402-406 (2016).
6. Durieux, P.F., *et al.* D2R striatopallidal neurons inhibit both locomotor and drug reward processes. *Nat Neurosci* **12**, 393-395 (2009).
7. Ena, S., de Kerchove d'Exaerde, A. & Schiffmann, S.N. Unraveling the differential functions and regulation of striatal neuron sub-populations in motor control, reward, and motivational processes. *Front Behav Neurosci* **5**, 47 (2011).
8. Bateup, H.S., *et al.* Distinct subclasses of medium spiny neurons differentially regulate striatal motor behaviors. *Proc Natl Acad Sci U S A* **107**, 14845-14850 (2010).
9. Carvalho Poyraz, F., *et al.* Decreasing Striatopallidal Pathway Function Enhances Motivation by Energizing the Initiation of Goal-Directed Action. *J Neurosci* **36**, 5988-6001 (2016).
10. Barbera, G., *et al.* Spatially Compact Neural Clusters in the Dorsal Striatum Encode Locomotion Relevant Information. *Neuron* **92**, 202-213 (2016).
11. Cui, G., *et al.* Concurrent activation of striatal direct and indirect pathways during action initiation. *Nature* **494**, 238-242 (2013).
12. Klaus, A., *et al.* The Spatiotemporal Organization of the Striatum Encodes Action Space. *Neuron* **95**, 1171-1180 e1177 (2017).
13. Weglage, M., *et al.* Complete representation of action space and value in all dorsal striatal pathways. *Cell Rep* **36**, 109437 (2021).
14. Markowitz, J.E., *et al.* The Striatum Organizes 3D Behavior via Moment-to-Moment Action Selection. *Cell* **174**, 44-58 e17 (2018).
