# Supplementary material for: The respective activation and silencing of striatal direct and indirect pathway neurons support behavior encoding
Source: Nat Commun. 2023 Aug 17;14:4982. doi: 10.1038/s41467-023-40677-0 (PMC10435545; doi:10.1038/s41467-023-40677-0)
Supplement: Supplementary file 3 — Reporting Summary [file 41467_2023_40677_MOESM3_ESM.pdf]

nature portfolio

Corresponding author(s): Alban de Kerchove d'Exaerde

Last updated by author(s): Jul 14, 2023

Reporting Summary

Nature Portfolio wishes to improve the reproducibility of the work that we publish. This form provides structure for consistency and transparency in reporting. For further information on Nature Portfolio policies, see our Editorial Policies and the Editorial Policy Checklist.

Statistics

For all statistical analyses, confirm that the following items are present in the figure legend, table legend, main text, or Methods section.

n/a	Confirmed	
☐	☒	The exact sample size (n) for each experimental group/condition, given as a discrete number and unit of measurement
☐	☒	A statement on whether measurements were taken from distinct samples or whether the same sample was measured repeatedly
☐	☒	The statistical test(s) used AND whether they are one- or two-sided<br>*Only common tests should be described solely by name; describe more complex techniques in the Methods section.*
☐	☒	A description of all covariates tested
☐	☒	A description of any assumptions or corrections, such as tests of normality and adjustment for multiple comparisons
☐	☒	A full description of the statistical parameters including central tendency (e.g. means) or other basic estimates (e.g. regression coefficient) AND variation (e.g. standard deviation) or associated estimates of uncertainty (e.g. confidence intervals)
☐	☒	For null hypothesis testing, the test statistic (e.g. F, t, r) with confidence intervals, effect sizes, degrees of freedom and P value noted<br>*Give P values as exact values whenever suitable.*
☒	☐	For Bayesian analysis, information on the choice of priors and Markov chain Monte Carlo settings
☒	☐	For hierarchical and complex designs, identification of the appropriate level for tests and full reporting of outcomes
☐	☒	Estimates of effect sizes (e.g. Cohen's d, Pearson's r), indicating how they were calculated

Our web collection on statistics for biologists contains articles on many of the points above.

Software and code

Policy information about availability of computer code

Data collection	Behavioral data was collected using Ethovision XT14 (Noldus). One-photon calcium videos were recorded using IDAS (Inscopix Data Acquisition Software versions 1.2.0 to 1.5.1; Inscopix).
Data analysis	For the analysis of behaviors, videos of mice in the open field were processed using DeepLabCut (Mathis et al., Nat Neurosci, 2018) to register a set of body parts. Subsequent identification of behaviors was performed using custom stripts in Matlab (Matlab 2018a, MathWorks). Primary analyses of calcium signal were performed using OpenCV (Python) for downscaling, CalmAn (Giovannucci et al., Elife, 2019) for motion correction and signal extraction, MLspike (Deneux et al., Nat Commun, 2016) for calcium signal deconvolution, and CellReg (Sheintuch et al., Cell Rep, 2017) for longitudinal registration of cell pairs.<br>All secondary analyses were performed using Matlab custom code, except support vector machine classification (scikit-learn package; Python). The custom codes used for this study are available on GitHub (https://github.com/deKerchove-Lab/Varin_2023_NatComm) with DOI (https://doi.org/10.5281/zenodo.8158538).

For manuscripts utilizing custom algorithms or software that are central to the research but not yet described in published literature, software must be made available to editors and reviewers. We strongly encourage code deposition in a community repository (e.g. GitHub). See the Nature Portfolio guidelines for submitting code & software for further information.

Data

Policy information about availability of data

All manuscripts must include a data availability statement. This statement should provide the following information, where applicable:

- Accession codes, unique identifiers, or web links for publicly available datasets
- A description of any restrictions on data availability
- For clinical datasets or third party data, please ensure that the statement adheres to our policy

The data supporting the findings are available within the article and its supplementary materials and are available from the corresponding author upon reasonable request. Source data are provided with this paper.

Research involving human participants, their data, or biological material

Policy information about studies with human participants or human data. See also policy information about sex, gender (identity/presentation), and sexual orientation and race, ethnicity and racism.

Reporting on sex and gender	NA
Reporting on race, ethnicity, or other socially relevant groupings	NA
Population characteristics	NA
Recruitment	NA
Ethics oversight	NA

Note that full information on the approval of the study protocol must also be provided in the manuscript.

Field-specific reporting

Please select the one below that is the best fit for your research. If you are not sure, read the appropriate sections before making your selection.

☒ Life sciences ☐ Behavioural & social sciences ☐ Ecological, evolutionary & environmental sciences

For a reference copy of the document with all sections, see nature.com/documents/nr-reporting-summary-flat.pdf

Life sciences study design

All studies must disclose on these points even when the disclosure is negative.

Sample size	No statistical measure was used to determine sample size. For all experiments, sample size was choosen according to standard practice in the field and our laboratory experience. Size was consistent with reports with similar experiments (Klaus et al., 2017, Neuron; Markowitz et al., 2018, Cell; Parker et al., 2018, Nature; Weglage et al., 2021, Cell Rep)
Data exclusions	Animals were excluded prior to data acquisition if the imaging quality or focal plane were poor or after acquisition but before secondary analyses if movement artifacts were impossible to correct using CalmAn.<br>After CNMF-E, the components were manually curated to remove components with poor signal-to-noise ratios (peak-to-noise ratio of less than around 3), large baseline fluctuations, or inappropriate spatial footprints.
Replication	Experiments were reproduced in 8 (Dl mice) and 9 (A2A mice) mice that were each recorded for 4-5 sessions. Attempts at reproduction were successful.
Randomization	Allocation of mice into experimental groups was not random as it relied on mice genotype. We controlled that behaviors expression in the open field is not different between the two experimental groups.<br>For saline or amphetamine injection experiments, saline administration occured randomly for any recording session but the last one, amphetamine treatment always occurred on the last day of recording to prevent any effect on neuronal activity due to the long-lasting effects of amphetamine.
Blinding	Data acquisition was performed blind to animal's genotype. Primary analyses (behaviors identification and extraction of calcium signal) were performed blind to the experimental condition.

Reporting for specific materials, systems and methods

We require information from authors about some types of materials, experimental systems and methods used in many studies. Here, indicate whether each material, system or method listed is relevant to your study. If you are not sure if a list item applies to your research, read the appropriate section before selecting a response.

Materials & experimental systems

n/a	Involved in the study
☒	☐ Antibodies
☒	☐ Eukaryotic cell lines
☒	☐ Palaeontology and archaeology
☐	☒ Animals and other organisms
☒	☐ Clinical data
☒	☐ Dual use research of concern
☒	☐ Plants

Methods

n/a	Involved in the study
☒	☐ ChIP-seq
☒	☐ Flow cytometry
☒	☐ MRI-based neuroimaging

Animals and other research organisms

Policy information about studies involving animals; ARRIVE guidelines recommended for reporting animal research, and Sex and Gender in Research

Laboratory animals	Three transgenic mouse lines were used: A2A-Cre, D1-Cre (EY262; GENSAT) and mice resulting from the crossing of A2A-Cre mice and Ai162/TIT2L-GC6s-ICL-tTA2 reporter line (Ai162; Allen Institute). The genetic background of all transgenic mice is C57Bl/6J. Both male and female mice were used in the study and were at least 8 weeks old at the start of experiments.
Wild animals	The study did not involve wild animals.
Reporting on sex	The study did not involve field-collected samples.
Field-collected samples	The study did not involve field-collected samples.
Ethics oversight	All procedures were performed according to the Institutional Animal Care Committee guidelines and were approved by the Local Ethical Committee (Comite d'Ethique et de Bien-Etre Animal du pole sante de l'Universite Libre de Bruxelles (ULB), Ref. No. 646 N).

Note that full information on the approval of the study protocol must also be provided in the manuscript.
